# Associations of polygenic risk scores for preeclampsia and blood pressure with hypertensive disorders of pregnancy

**DOI:** 10.1097/HJH.0000000000003336

**Published:** 2022-12-16

**Authors:** Jouko Nurkkala, Anni Kauko, FinnGen, Hannele Laivuori, Tanja Saarela, Jaakko S. Tyrmi, Felix Vaura, Susan Cheng, Natalie A. Bello, Jenni Aittokallio, Teemu Niiranen

**Affiliations:** a Division of Perioperative Services, Intensive Care and Pain Medicine, Turku University Hospital; b Department of Anesthesiology and Intensive Care; c Department of Internal Medicine, University of Turku, Turku; d Department of Obstetrics and Gynecology, Tampere University Hospital; e Center for Child, Adolescent, and Maternal Health Research, Faculty of Medicine and Health Technology, Tampere University, Tampere; f Institute for Molecular Medicine Finland (FIMM), Helsinki Institute of Life Science, University of Helsinki; g Medical and Clinical Genetics, University of Helsinki and Helsinki University Hospital, Helsinki; h Department of Clinical Genetics, Kuopio University Hospital, Kuopio; i Faculty of Medicine and Health Technology, University of Tampere, Tampere, Finland; j Smidt Heart Institute, Cedars-Sinai Medical Center, Los Angeles, California; k Division of Cardiology Brigham and Women's Hospital, Boston, Massachusetts, USA; l Division of Medicine, Turku University Hospital; m Department of Public Health Solutions, Finnish Institute for Health and Welfare, Turku, Finland

**Keywords:** blood pressure, genetics, hypertension, polymorphism, preeclampsia, pregnancy-induced, risk factors, single nucleotide

## Abstract

**Background:**

Preexisting hypertension increases risk for preeclampsia. We examined whether a generic blood pressure polygenic risk score (BP-PRS), compared with a preeclampsia-specific polygenic risk score (PE-PRS), could better predict hypertensive disorders of pregnancy.

**Methods:**

Our study sample included 141 298 genotyped FinnGen study participants with at least one childbirth and followed from 1969 to 2021. We calculated PRSs for SBP and preeclampsia using summary statistics for greater than 1.1 million single nucleotide polymorphisms.

**Results:**

We observed 8488 cases of gestational hypertension (GHT) and 6643 cases of preeclampsia. BP-PRS was associated with GHT [multivariable-adjusted hazard ratio for 1SD increase in PRS (hazard ratio 1.38; 95% CI 1.35–1.41)] and preeclampsia (1.26, 1.23–1.29), respectively. The PE-PRS was also associated with GHT (1.16; 1.14–1.19) and preeclampsia (1.21, 1.18–1.24), but with statistically more modest magnitudes of effect (*P* = 0.01). The model c-statistic for preeclampsia improved when PE-PRS was added to clinical risk factors (*P* = 4.6 × 10^–15^). Additional increment in the c-statistic was observed when BP-PRS was added to a model already including both clinical risk factors and PE-PRS (*P* = 1.1 × 10^–14^).

**Conclusion:**

BP-PRS is strongly associated with hypertensive disorders of pregnancy. Our current observations suggest that the BP-PRS could capture the genetic architecture of preeclampsia better than the current PE-PRSs. These findings also emphasize the common pathways in the development of all BP disorders. The clinical utility of a BP-PRS for preeclampsia prediction warrants further investigation.

## INTRODUCTION

Preeclampsia is a leading cause of maternal and fetal morbidity and mortality worldwide, especially in low-income countries [1,2]. Preeclampsia is characterized by *de novo* hypertension during pregnancy after 20 weeks of gestation with proteinuria or other end-organ complications [3]. In women with chronic hypertension, which precedes pregnancy or develops prior to 20 weeks of gestation, superimposed preeclampsia can occur. Apart from these more serious conditions, new onset of hypertension in pregnancy without proteinuria is more common and is referred to as gestational hypertension (GHT). Chronic hypertension during pregnancy, GHT, preeclampsia, chronic hypertension with superimposed preeclampsia and eclampsia are collectively referred to as hypertensive disorders of pregnancy [3].

Considerable overlap and fluidity of diagnoses exist between various hypertensive disorders of pregnancy. For example, chronic hypertension diagnosed before pregnancy is an independent risk factor for preeclampsia [4]. On the other hand, preeclampsia is associated with an increased risk of chronic hypertension and cardiovascular disease later in life [5,6]. Prior data suggest that this overlap may be partly driven by genetic factors, as chronic hypertension has been associated with several autosomal single nucleotide polymorphisms (SNPs) [7]. A recent meta-analysis on genome-wide association studies (GWASs) for preeclampsia also identified five maternal genetic variants previously associated with chronic hypertension [8]. In addition, a polygenic risk score (PRS) for hypertension was associated with an increased risk of preeclampsia [8]. In a recent study by Kivioja *et al.*[9], a high BP-PRS score was associated with preeclampsia and especially its more severe forms. However, the associations between genetic propensity for chronic hypertension and other hypertensive disorders of pregnancy disorders remain unknown. In addition, the added predictive value of a blood pressure-specific PRS (BP-PRS) versus a preeclampsia-specific PRS (PE-PRS) is unclear.

Our aim was to examine the common genetic background between chronic hypertension and hypertensive disorders of pregnancy. In this study, we investigated the association of a blood pressure-specific PRS (BP-PRS); preeclampsia-specific PRS (PE-PRS); and clinical risk factors with hypertensive disorders of pregnancy. We also compared the predictive ability of these risk factors in more than 140 000 previously pregnant FinnGen study participants.

## METHODS

### Study sample

Our cohort study sample consisted of 210 870 genotyped Finnish women from the FinnGen Data Freeze 9, which included participants from Finnish cohort studies and patients from national hospital biobanks [10]. Of these, 141 298 had given birth and were selected for further analysis. All participants provided written informed consent. This study protocol was approved by The Coordinating Ethical Committee of the Hospital District of Helsinki and Uusimaa, as described in the Supplemental Methods.

Because of the sensitive nature of the data collected for this study, requests to access the dataset from qualified researchers trained in human subject confidentiality protocols may be submitted through the Finnish Biobanks’ Finngenious portal (https://site.fingenious.fi/en/) for longitudinal and genetic data.

### Genotyping and polygenic risk scores

The collected DNA samples in FinnGen study were genotyped with Illumina (Illumina Inc., San Diego, California, USA) and Affymetrix (Thermo Fisher Scientific, Santa Clara, California, USA) arrays and genotype calls were made with zCall or GenCall algorithms (for Illumina) and AxiomGT1 algorithm (for Affymetrix) at the Institute for Molecular Medicine Finland (FIMM). Quality control exclusions were performed first sample-wise: ambiguous gender, high genotype missingness greater than 5%, excess heterozygosity greater than ±4SD, or non-European ancestry; and second, variant-wise: missingness greater than 2%, low Hardy–Weinberg equilibrium *P* less than 1 × 10^−6^, minor allele count less than 3, were excluded. After quality control, the samples were prephased with Eagle 2.3.5 with default parameters and then genotypes were imputated with Beagle 4.1 (version 08Jun17.d8b) using a Finnish population-specific SISu v3 reference panel. Finally, to account for population structure in downstream analyses, genetic principal component analysis (PCA) was performed using a pruned set of SNPs of unrelated individuals. Detailed documentation of genotyping, imputation, and principal component analysis is available online [11].

BP-PRSs and PE-PRS were computed using the PRS-CS [12] pipeline with default parameters. PRS-CS computes SNP effect sizes by high-dimensional Bayesian regression with continuous shrinkage priors using the obtained GWAS summary statistics and a linkage disequilibrium reference panel. The summary statistics for SBP was obtained from UK Biobank [13,14] and it was based on 182 645 women. Preeclampsia summary statistics were obtained from previously published meta-analysis made by Genetics of Pre-Eclampsia Consortium [8]. However, to avoid potential overfitting because of Finnish participants in the GWAS, we obtained GWAS summary statistics from the authors of this report without the Finnish individuals included. Thus, the summary statistics for preeclampsia was based on 9115 cases of preeclampsia and 149 914 controls. The European linkage disequilibrium reference panel with 1.1 million variants was derived from samples of the 1000 Genomes Project [15]. The BP-PRS and PE-PRSs were based on 1 098 015 genetic variants common in the linkage disequilibrium reference panel and FinnGen.

### Register-based outcomes

The calculated individual-level PRSs were linked to register-based predictors and outcomes using personal national identification codes. Every Finnish permanent resident is linked to National Hospital Discharge (from 1968) and Cause of Death (from 1969) Registers, which makes follow-up possible for all major clinical end points, including preeclampsia and GHT. Birth data was obtained from the Medical Birth and Population Information Registers, while disease events were retrieved from the Hospital Discharge and Causes-of-death Registers. The quality of the diagnoses in the Hospital Discharge and Causes-of-Death Registers is good and has been described in detail previously [16]. The clinical diagnoses in the registers are based on ICD codes made by the attending primary or secondary care physician and the definitions of the diagnoses used in this study are described in detail in the Supplementary Table S1. The following outcomes were used: GHT and preeclampsia. In analyses that assessed improvement in model fit, the variables included in the clinical model (age, obesity, hypertension, diabetes, gestational diabetes, multifetal pregnancy, in-vitro fertilization pregnancies, and renal insufficiency) were drawn from the same registers (Supplementary Table S1).

### Statistical analyses

We used Cox proportional hazards model to assess the association between a 1SD increase in BP-PRS or PE-PRS and the outcomes. We also performed a sensitivity analysis by analyzing the individuals with and without a diagnosis of hypertension prior to the index pregnancy separately. The magnitudes of associations between different PRSs and outcomes were compared with Student's *t* test. The follow-up spanned from 1969 to 2021. An individual participant was censored only once at the first encountered episode of preeclampsia or GHT. Both cases and controls were censored at death or at the end of follow-up (age 55 or 11 October 2021). Age was considered as the timescale and we used collection year, genotyping batch, and the first 10 genetic principal components as covariates in all models. The proportional hazards assumption was validated by visual inspection of log-minus-log plots because of the large sample size.

Furthermore, we categorized the participants by their PRS-count percentiles (<2.5, 2.5–20, 20–80, 80–97.5, >97.5) and denoted the middle bin (20–80%) as the referent category. We then used Cox proportional hazards model for the four remaining categories to investigate the associations for preeclampsia and GHT using mother age as a timescale.

To include age at pregnancy as a covariate, we used logistic regression-based c-statistic to compare the predictive ability of three alternative models for predicting preeclampsia: clinical model, which included age at pregnancy, plus the following variables known to associate with preeclampsia: obesity, hypertension, diabetes, gestational diabetes, multifetal pregnancy, in-vitro fertilization, and renal insufficiency [17]; clinical model + PE-PRS; and clinical model + PE-PRS + BP-PRS. Previous preeclampsia was not included in the clinical model as we focused on the risk of first preeclampsia. Family history of preeclampsia was not included in the clinical model as this information was not available. All covariates included in the clinical model were observed before the outcome event of the index pregnancy. Also, pregnancy-related covariates were linked with the index pregnancy.

We also calculated the net reclassification index (NRI) and the integrated discrimination index (IDI) to further assess improvement in reclassification and risk discrimination. We used an 8% risk threshold for the categorical NRI, consistent with the lowest preeclampsia incidence observed in control groups in studies reviewed by a recent US Preventive Services Task Force Recommendation Statement [18]. Further, we assessed the variance explained by the models by calculating the pseudo-R-squared with the McKelvey–Zavoina method. We considered two-tailed *P* values of 0.05 as statistically significant and used R v.4.2.1 for all analyses.

## RESULTS

Our study sample consisted of 141 298 women with childbirth with a mean age of 27.1 ± 5.2 years at time of first delivery. The characteristics of the study sample are shown in Table 1. We observed 8488 cases (6.1%) of GHT and 6643 cases (4.9%) of preeclampsia.

**TABLE 1:** The characteristics of the study sample by outcome status

	Gestational hypertension		Preeclampsia	
Characteristic	Yes	No	Yes	No
*n*	8488	129 829	6643	129 711
Age (mean ± SD)	30.1 ± 5.7	27.1 ± 5.1	29.1 ± 5.7	27.1 ± 5.1
Multifetal pregnancy [*n* (%)]	216 (2.5)	1581 (1.2)	288 (4.3)	1553 (1.2)
IVF [*n* (%)]	16 (0.2)	163 (0.1)	11 (0.2)	163 (0.1)
Hypertension [*n* (%)]	273 (3.2)	470 (0.4)	172 (2.6)	495 (0.4)
Obesity [*n* (%)]	310 (3.7)	891 (0.7)	166 (2.5)	897 (0.7)
Diabetes [*n* (%)]	1186 (14.0)	5633 (4.4)	904 (13.6)	5616 (4.3)
Renal failure [*n* (%)]	8 (0.1)	38 (0.03)	8 (0.1)	38 (0.03)

Characteristics for the controls are reported at the time of first pregnancy. Gestational diabetes is included to the diabetes. IVF, in-vitro fertilization.

Both BP-PRS and PE-PRS were associated with GHT and preeclampsia (Table 2). For the BP-PRS, the hazard ratios for GHT and preeclampsia were 1.38 (95% CI 1.35–1.41; *P* = 8.8 × 10^–194^) and 1.26 (95% CI 1.23–1.29; *P* = 3.5 × 10^−78^), respectively. For PE-PRS, the corresponding hazard ratios were more modest – 1.16 (95% CI 1.14–1.19; *P* = 1.5 × 10^−44^), and 1.21 (95% CI 1.18–1.24; *P* = 1.2 × 10^–55^), respectively. The associations of BP-PRS with GHT and preeclampsia was stronger than those observed between PE-PRS and the clinical outcomes (*P* < 0.01 for all).

**TABLE 2:** Association of polygenic risk scores for blood pressure with hypertensive disorders of pregnancy

			Blood pressure PRS		Preeclampsia PRS		
Endpoint	Cases	Controls	HR (95% CI)	*P* value	HR (95% CI)	*P* value	*P* value for HR difference
Gestational hypertension	8488	129 829	1.38 (1.35–1.41)	8.8 × 10^−194^	1.16 (1.14–1.19)	1.5 × 10^−44^	1.7 × 10^−31^
Preeclampsia	6643	129 711	1.26 (1.23–1.29)	3.5 × 10^−78^	1.21 (1.18–1.24)	1.2 × 10^−55^	0.01

We adjusted the models for collection year, genotyping batch, and the first 10 genetic principal components. Hazard ratios are reported per 1SD increment in polygenic risk score. CI, confidence interval; HR, hazard ratio; PRS, polygenic risk score.

We also performed the analyses separately for individuals with and without a diagnosis of hypertension prior to the index pregnancy. We observed that the BP-PRS was associated with future eclampsia among the 135 687 individuals without a diagnosis of hypertension [6471 cases; hazard ratio 1.26 (95% CI 1.23–1.29)], but not among the 667 individuals who had hypertension [172 cases; hazard ratio 1.01 (95% CI 0.88–1.17)]. The results were similar for PE-PRS [hazard ratio 1.21 (95% CI 1.18–1.24) vs. hazard ratio 1.10 (95% CI 0.94–1.29)].

**FIGURE 1:**
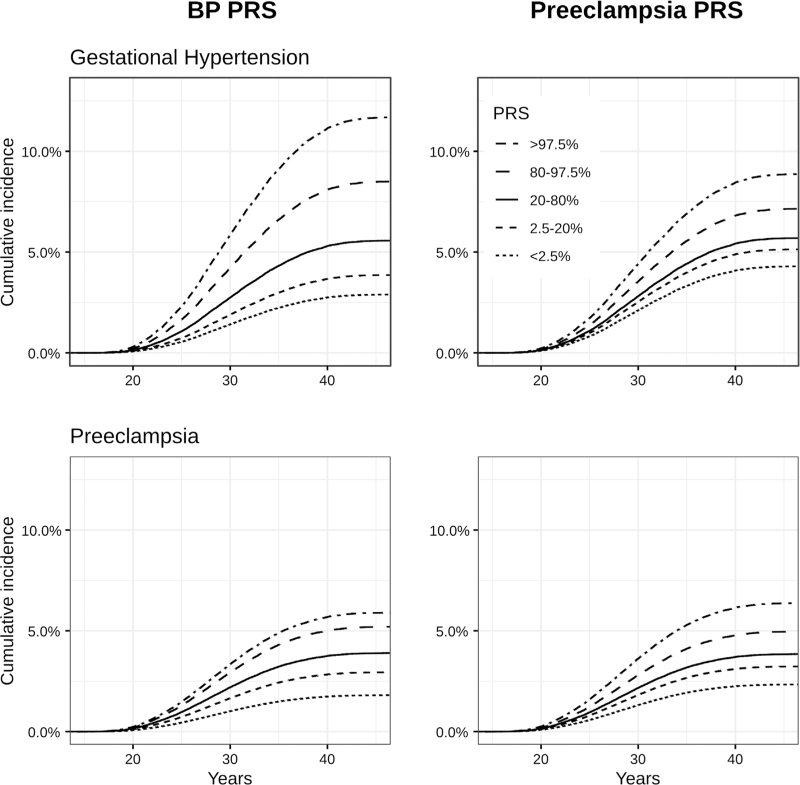
Cumulative incidence of hypertensive disorders of pregnancy by blood pressure and preeclampsia polygenic risk score bins. The survival curves are from Cox proportional hazards models. We adjusted the models for collection year, genotyping batch and the first 10 genetic principal components. BP, blood pressure; PRS, polygenic risk score.

The analyses examining the association of PRS quantiles and GHT and preeclampsia are reported in Fig. 1 and Table 3. For GHT, the spread of hazard ratios between the low-risk and high-risk categories was wider when BP-PRS category was used as the exposure variable, as compared with the PE-PRS category (Fig. 1).

**TABLE 3:** Risk of hypertensive disorders of pregnancy by blood pressure and preeclampsia polygenic risk score bins

	Blood pressure PRS				Preeclampsia PRS			
PRS (%)	Cases	Controls	HR (95% CI)	*P* value	Cases	Controls	HR (95% CI)	*P* value
Gestational hypertension								
<2.5%	103	3369	0.51 (0.42–0.62)	1.7 × 10^−11^	151	3319	0.75 (0.64–0.88)	4.2 × 10^–4^
2.5–20%	974	23 418	0.69 (0.64–0.74)	1.2 × 10^−26^	1288	23 026	0.90 (0.85–0.96)	6.4 × 10^–4^
20–80%	4815	77 990	–	–	4913	78 031	–	–
80–97.5%	2169	22 034	1.55 (1.47–1.63)	3.4 × 10^−64^	1814	22 323	1.26 (1.20 -1.33)	1.3 × 10^−17^
>97.5%	427	3018	2.17 (1.96–2.39)	7.8 × 10^−53^	322	3130	1.58 (1.41–1.77)	1.4 × 10^−15^
Preeclampsia								
<2.5%	77	3368	0.46 (0.37–0.57)	1.3 × 10^−11^	98	3335	0.60 (0.49–0.74)	7.7 × 10^–7^
2.5–20%	878	23 344	0.75 (0.70–0.81)	2.2 × 10^−14^	954	23 049	0.84 (0.78–0.90)	9.2 × 10^–7^
20–80%	3928	77 819	–	–	3867	77 900	–	–
80–97.5%	1518	22 110	1.34 (1.27–1.43)	1.7 × 10^−22^	1456	22 311	1.30 (1.22–1.38)	2.1 × 10^−17^
>97.5%	242	3070	1.53 (1.35–1.74)	1.3 × 10^−10^	268	3116	1.68 (1.49–1.90)	2.0 × 10^−16^

CI, confidence interval; HR, hazard ratio; PRS, polygenic risk score. We adjusted the models for collection year, genotyping batch, and the first 10 genetic principal components.

We then assessed the improvements in risk discrimination and reclassification when the PE-PRS and/or the BP-PRS were included in a model with known clinical preeclampsia risk factors (Table 4). The addition of PE-PRS in the clinical model significantly increased the c-statistic (c-statistic increment 0.015; 95% CI 0.011–0.018; *P* = 4.6 × 10^–15^). However, including the BP-PRS among the predictor variables resulted in an additional improvement in the c-statistic (0.013; 95% CI 0.001–0.017, *P* = 1.1 × 10^–14^). Also, the strength of the associations of both PRSs with preeclampsia remained similar when clinical covariates were included in the same model (Table S2). The NRI and IDI increased significantly (*P* = 4.5 × 10^–5^ and 6.2 × 10^–32^, respectively) when the PE-PRS was included in the clinical model. However, additional increase was observed in NRI and IDI when BP-PRS (NRI = 0.023, *P* = 7.8 × 10^–13^, IDI = 0.0029, *P* = 4.1 × 10^–41^) was added in the model that included the clinical variables and the PE-PRS. Variances of the Cox models assessed with the pseudo-*R*^2^ were 5.5% for the clinical model, 6.6% for the clinical + PE-PRS -model and 8% for the clinical + PE-PRS + BP-PRS model (Table 4).

**TABLE 4:** Model fit, calibration, and discrimination statistics for different preeclampsia risk prediction models

	c-statistic			NRI				IDI		
Model	c-statistic	Increment (95% CI)	*P* value	NRI (95% CI)	*P* value	Correctly reclassified cases (%)	Correctly reclassified controls (%)	IDI (95% CI)	*P* value	Pseudo *R*^2^ (%)
Clinical model^a^	0.634									5.5
Clinical model + preeclampsia PRS	0.649	0.015 (0.011–0.018)	4.6 × 10^−15^	0.011 (0.006–0.017)	4.5 × 10^–5^	4.3	11.6	0.0022 (0.0018–0.0025)	6.2 × 10^−32^	6.6
Clinical model + preeclampsia PRS + blood pressure PRS	0.661	0.013 (0.001–0.017)	1.1 × 10^−14^	0.023 (0.017–0.029)	7.8 × 10^−13^	6.5	15.2	0.0029 (0.0024–0.0033)	4.1 × 10^−41^	8.0

CI, confidence interval; IDI, integrated discrimination index; NRI, net reclassification index; PRS, polygenic risk score.

a Model includes age, hypertension, obesity, diabetes, gestational diabetes, multifetal pregnancies, in-vitro fertilization and renal failure. NRI cut off was set at 8%.

## DISCUSSION

In a study sample of 141 298 women with childbirth, we demonstrate that BP-PRS is strongly associated with preeclampsia and that this association is even stronger for GHT (Table 2). Our current observations suggest a strong common genetic background for chronic hypertension, preeclampsia, and hypertensive disorders of pregnancy.

Epidemiological studies have demonstrated that maternal, paternal, and fetal components of the genetic susceptibility for preeclampsia exist [19,20]. Our results elucidate that this susceptibility may be partially driven by BP, a strongly polygenic trait [21–28]. The relation between high BP and hypertensive disorders of pregnancy has also been observed in epidemiological studies in which chronic hypertension diagnosed before pregnancy was related to five-fold increase in the risk of preeclampsia [4,17].

Prior maternal GWASs on preeclampsia have discovered associations with several genetic variants that are also associated with BP [8,29]. Moreover, an association between a PRS for hypertension and preeclampsia has also been formerly identified by a meta-analysis of 9515 preeclamptic women and 157 719 controls from five cohorts [8]. In a recent smaller study of 1514 preeclamptic individuals and 983 controls, a BP-PRS above the 95th percentile was associated with 1.7-fold greater odds of preeclampsia and especially its more severe forms. However, this association was not statistically significant after adjustment for the first antenatal blood pressure measurement [9]. This risk increase is similar to that observed for a BP-PRS in the top 2.5 percentile in our study. However, over 95% of individuals in our study were normotensive before the index pregnancy. Our results expand these prior results by providing a comprehensive analysis on the associations between a BP-PRS and hypertensive disorders of pregnancy in a relatively unselected cohort over a follow-up period spanning 50 years.

The exact mechanism of the observed common genetic background for preeclampsia, chronic hypertension, and future cardiovascular disease (CVD) risk remains still unclear. Preeclampsia is an independent risk factor for several cardiovascular diseases later in life including chronic hypertension, stroke, coronary artery disease, and heart failure [5,30–32]. However, it is debated, whether the defective placentation followed by maternal manifestations leads to permanent alterations in the vasculature and increased CVD risk, or whether there are an underlying maternal susceptibility for preeclampsia, hypertension, and CVD that is unveiled by the pregnancy [33–35]. It is clear that several pathogenetic mechanisms of preeclampsia exist apart from genetic hypertension risk, such as defective spiral artery modelling, systemic inflammation, and immunologic response [36]. These mechanisms may also differ between early and late preeclampsia [36]. However, as the clinical [17], epidemiological [19], and genetic links [8,29,36] between preeclampsia and hypertension have been demonstrated, hypertension is clearly one of the major contributors to risk of preeclampsia. Our study with BP-PRS enforces the approach that there is a preexisting maternal genetic influence behind both preeclampsia and chronic hypertension with the latter being known, strong risk factor for CVD.

Compared with PE-PRS, the association between BP-PRS and hypertensive disorders of pregnancy was stronger. Several prior GWASs have identified genetic variants related to preeclampsia [29,37–40], but genetic risk scores based on preeclampsia-related SNPs have produced only modest results [41,42]. In this article, we demonstrate that using a BP-PRS to predict hypertensive disorders of pregnancy is equal or even better than conventional approach of using a PE-PRS as the predictor. However, these findings applied only to women who were nonhypertensive before the index pregnancy. Possible explanations on the lack of associations in the hypertensive individuals include the low number of individuals with hypertension preceding pregnancy and the confounding effects of antihypertensive therapy among the hypertensive individuals [43,44].

Further, our results in c-statistic, NRI, IDI, and *R*^2^ demonstrate that the improvement in risk prediction and discrimination of preeclampsia is only marginal when a PE-PRS is used (Table 4). In addition, an additional improvement in all these indices was observed when BP-PRS was included, even when a diagnoses of prior hypertension and other clinical risk factors were already included in the model. Thus, BP-PRS could, therefore, be potentially used in clinical practice for assessing risk of preeclampsia.

Despite the strengths of our study, such as a large sample size, a standardized and reliable method for assessing a wide range of clinical outcomes, and application of a PRS derived from over 1.1 million SNPs, the results of our study must be interpreted within the context of potential limitations. First, we used outcome data from Finnish nationwide healthcare registers, which are generally complete and accurate, but may nevertheless lack the granularity of detailed clinical data [16]. However, this potential limitation is similar for both BP-PRS and PE-PRS and is, therefore, unlikely to have a major effect on our findings. Second, as in most GWAS, we analyzed only autosomal SNP variants, which may omit important genetic information from the sex chromosomes. Third, given that our sample constituted mainly of individuals of Northern European ancestry, further studies are needed to determine generalizability of findings to other groups. Fourth, some relevant preeclampsia risk factors, such as family history of preeclampsia, inflammation indices, and glomerular filtration rate were not available in the FinnGen data. Similarly, the prevalence of cardiovascular disease was too low for it to be included in the statistical models. Finally, as the diagnoses of hypertension and preeclampsia in our study are register-based and span five decades, we do not have information on the exact blood pressure measurement methods for each individual.

### Perspectives

In conclusion, we demonstrate in a sample of more than 140 000 previously pregnant women that genetic autosomal polymorphism related to BP is strongly associated with increased risk of preeclampsia and gestational hypertension. In addition, this association appears to be stronger than what is observed for PE-PRS and preeclampsia. The exact mechanisms of this dual effect on the development of essential hypertension and preeclampsia and increased risk of cardiovascular disease later in life are unknown, but may point to a common pathway of development, which warrants further investigation. Furthermore, prospective trials are needed to evaluate the value of BP-PRSs as a clinical screening tool and to define clinically significant cutoff values for BP-PRS.

## ACKNOWLEDGEMENTS

We want to acknowledge the participants and investigators of FinnGen study. Following biobanks are acknowledged for delivering biobank samples to FinnGen: Auria Biobank (www.auria.fi/biopankki), THL Biobank (www.thl.fi/biobank), Helsinki Biobank (www.helsinginbiopankki.fi), Biobank Borealis of Northern Finland (https://www.ppshp.fi/Tutkimus-ja-opetus/Biopankki/Pages/Biobank-Borealis-briefly-in-English.aspx), Finnish Clinical Biobank Tampere (www.tays.fi/en-US/Research_and_development/Finnish_Clinical_Biobank_Tampere), Biobank of Eastern Finland (www.ita-suomenbiopankki.fi/en), Central Finland Biobank (www.ksshp.fi/fi-FI/Potilaalle/Biopankki), Finnish Red Cross Blood Service Biobank (www.veripalvelu.fi/verenluovutus/biopankkitoiminta), and Terveystalo Biobank (www.terveystalo.com/fi/Yritystietoa/Terveystalo-Biopankki/Biopankki/). All Finnish Biobanks are members of BBMRI.fi infrastructure (www.bbmri.fi). Finnish Biobank Cooperative -FINBB (https://site.fingenious.fi/en/) is the coordinator of BBMRI-ERIC operations in Finland. The Finnish biobank data can be accessed through the Fingenious services (https://site.fingenious.fi/en/) managed by FINBB.

Sources of funding: The FinnGen project is funded by two grants from Business Finland (HUS 4685/31/2016 and UH 4386/31/2016) and the following industry partners: AbbVie Inc., AstraZeneca UK Ltd, Biogen MA Inc., Bristol Myers Squibb (and Celgene Corporation & Celgene International II Sàrl), Genentech Inc., Merck Sharp & Dohme Corp, Pfizer Inc., GlaxoSmithKline Intellectual Property Development Ltd., Sanofi US Services Inc., Maze Therapeutics Inc., Janssen Biotech Inc, Novartis AG, and Boehringer Ingelheim. S.C. is funded by NIH grant U54-AG065141. N.B. is funded by NIH/NHLBI grant K23 HL136853 and R01 HL153382. J.A. is funded by the Finnish Medical Foundation and the State Research Funds of the Hospital District of Southwest Finland. T.N. is funded by the Academy of Finland (grant n:o 321351), the Sigrid Juselius Foundation, and the Finnish Foundation for Cardiovascular Research.

### Conflicts of interest

There are no conflicts of interest.

## Supplementary Material

Supplemental Digital Content

## References

[1] Duley L. The global impact of preeclampsia and eclampsia. *Semin Perinatol* 2009; 33:130–137. 19464502 10.1053/j.semperi.2009.02.010

[2] Abalos E Cuesta C Grosso AL Chou D Say L. Global and regional estimates of preeclampsia and eclampsia: a systematic review. *Eur J Obstet Gynecol Reprod Biol* 2013; 170:1–7. 23746796 10.1016/j.ejogrb.2013.05.005

[3] Hypertension in pregnancy. Report of the American College of Obstetricians and Gynecologists’ Task Force on Hypertension in Pregnancy. *Obstet Gynecol* 2013; 122:1122–1131. 24150027 10.1097/01.AOG.0000437382.03963.88

[4] Bartsch E Medcalf KE Park AL Ray JG. Group HR of PI. Clinical risk factors for preeclampsia determined in early pregnancy: systematic review and meta-analysis of large cohort studies. *BMJ* 2016; 353:i1753–i11753. 27094586 10.1136/bmj.i1753 PMC4837230

[5] Bellamy L Casas J-PP Hingorani AD Williams DJ. Preeclampsia and risk of cardiovascular disease and cancer in later life: systematic review and meta-analysis. *BMJ* 2007; 335:974. 17975258 10.1136/bmj.39335.385301.BE PMC2072042

[6] Brouwers L van der Meiden-van Roest AJ Savelkoul C Vogelvang TE Lely AT Franx A. Recurrence of preeclampsia and the risk of future hypertension and cardiovascular disease: a systematic review and meta-analysis. *BJOG* 2018; 125:1642–1654. 29978553 10.1111/1471-0528.15394 PMC6283049

[7] Giri A Hellwege JN Keaton JM Park J Qiu C Warren HR. Trans-ethnic association study of blood pressure determinants in over 750,000 individuals. *Nat Genet* 2019; 51:51–62. 30578418 10.1038/s41588-018-0303-9 PMC6365102

[8] Steinthorsdottir V McGinnis R Williams NO Stefansdottir L Thorleifsson G Shooter S. Genetic predisposition to hypertension is associated with preeclampsia in European and Central Asian women. *Nat Commun* 2020; 11:5976. 33239696 10.1038/s41467-020-19733-6 PMC7688949

[9] Kivioja A Toivonen E Tyrmi J Ruotsalainen S Ripatti S Huhtala H. Increased risk of preeclampsia in women with a genetic predisposition to elevated blood pressure. *Hypertension* 2022; 79:2008–2015. 35862124 10.1161/HYPERTENSIONAHA.122.18996 PMC9370253

[10] Mars N Koskela JT Ripatti P Kiiskinen TTJ Havulinna AS Lindbohm JV. Polygenic and clinical risk scores and their impact on age at onset and prediction of cardiometabolic diseases and common cancers. *Nat Med* 2020; 26:549–557. 32273609 10.1038/s41591-020-0800-0

[11] FinnGen. FinnGen documentation of R7 release available at: https://finngen.gitbook.io/documentation/v/r7/

[12] Ge T Chen C-Y Ni Y Feng Y-CA Smoller JW. Polygenic prediction via Bayesian regression and continuous shrinkage priors. *Nat Commun* 2019; 10:1776. 30992449 10.1038/s41467-019-09718-5 PMC6467998

[13] UK Biobank Neale-lab website. GWAS round 2 results. Available at: http://www.nealelab.is/uk-biobank.

[14] Sudlow C Gallacher J Allen N Beral V Burton P Danesh J. UK biobank: an open access resource for identifying the causes of a wide range of complex diseases of middle and old age. *PLoS Med* 2015; 12:e1001779. 25826379 10.1371/journal.pmed.1001779 PMC4380465

[15] Auton A Abecasis GR Altshuler DM Durbin RM Bentley DR Chakravarti A. A global reference for human genetic variation. *Nature* 2015; 526:68–74. 26432245 10.1038/nature15393 PMC4750478

[16] Sund R. Quality of the Finnish Hospital Discharge Register: a systematic review. *Scand J Public Health* 2012; 40:505–515. 22899561 10.1177/1403494812456637

[17] Chappell LC Cluver CA Kingdom J Tong S. Preeclampsia. *Lancet* 2021; 398:341–354. 34051884 10.1016/S0140-6736(20)32335-7

[18] Henderson JT, Vesco KK, Senger CA *et al.* aspirin use to prevent preeclampsia and related morbidity and mortality: an evidence update for the U.S. Preventive Services Task Force. Evidence Synthesis No. 205.

[19] Cnattingius S Reilly M Pawitan Y Lichtenstein P. Maternal and fetal genetic factors account for most of familial aggregation of preeclampsia: a population-based Swedish cohort study. *Am J Med Genet A* 2004; 130A:365–371. 15384082 10.1002/ajmg.a.30257

[20] Esplin MS Fausett MB Fraser A Kerber R Mineau G Carrillo J. Paternal and maternal components of the predisposition to preeclampsia. *N Engl J Med* 2001; 344:867–872. 11259719 10.1056/NEJM200103223441201

[21] Ehret GB Munroe PB Rice KM Bochud M Johnson AD. International Consortium for Blood Pressure Genome-Wide Association Studies. Genetic variants in novel pathways influence blood pressure and cardiovascular disease risk. *Nature* 2011; 478:103–109. 21909115 10.1038/nature10405 PMC3340926

[22] Warren HR Evangelou E Cabrera CP Gao H Ren M Mifsud B. International Consortium of Blood Pressure (ICBP) 1000G Analyses, BIOS Consortium, Lifelines Cohort Study, Understanding Society Scientific group, CHD Exome+ Consortium, ExomeBP Consortium, T2D-GENES Consortium, GoT2DGenes Consortium, Cohorts for Heart and Ageing Research in Genome Epidemiology (CHARGE) BP Exome Consortium, International Genomics of Blood Pressure (iGEN-BP) Consortium, UK Biobank CardioMetabolic Consortium BP working group. Genome-wide association analysis identifies novel blood pressure loci and offers biological insights into cardiovascular risk. *Nat Genet* 2017; 49:403–415.28135244

[23] Fava C Sjögren M Montagnana M Danese E Almgren P Engström G. Prediction of blood pressure changes over time and incidence of hypertension by a genetic risk score in Swedes. *Hypertension* 2013; 61:319–326. 23232644 10.1161/HYPERTENSIONAHA.112.202655

[24] Niiranen TJ Havulinna AS Langén VL Salomaa V Jula AM. Prediction of blood pressure and blood pressure change with a genetic risk score. *J Clin Hypertens (Greenwich)* 2016; 18:181–186. 26435379 10.1111/jch.12702 PMC8032027

[25] Lim N-K Lee J-Y Lee J-Y Park H-Y Cho M-C. The role of genetic risk score in predicting the risk of hypertension in the Korean population: Korean Genome and Epidemiology Study. *PLoS One* 2015; 10:e0131603. 26110887 10.1371/journal.pone.0131603 PMC4482533

[26] Oikonen M Tikkanen E Juhola J Tuovinen T Seppälä I Juonala M. Genetic variants and blood pressure in a population-based cohort: the Cardiovascular Risk in Young Finns study. *Hypertension* 2011; 58:1079–1085. 22025373 10.1161/HYPERTENSIONAHA.111.179291 PMC3247907

[27] Juhola J Oikonen M Magnussen CG Mikkilä V Siitonen N Jokinen E. Childhood physical, environmental, and genetic predictors of adult hypertension: the cardiovascular risk in young Finns study. *Circulation* 2012; 126:402–409. 22718800 10.1161/CIRCULATIONAHA.111.085977

[28] Havulinna AS Kettunen J Ukkola O Osmond C Eriksson JG Kesäniemi YA. A blood pressure genetic risk score is a significant predictor of incident cardiovascular events in 32,669 individuals. *Hypertension* 2013; 61:987–994. 23509078 10.1161/HYPERTENSIONAHA.111.00649 PMC3648219

[29] Gray KJ Kovacheva VP Mirzakhani H Bjonnes AC Almoguera B DeWan AT. Gene-centric analysis of preeclampsia identifies maternal association at PLEKHG1. *Hypertension* 2018; 72:408–416. 29967039 10.1161/HYPERTENSIONAHA.117.10688 PMC6043396

[30] Brown MC Best KE Pearce MS Waugh J Robson SC Bell R. Cardiovascular disease risk in women with preeclampsia: systematic review and meta-analysis. *Eur J Epidemiol* 2013; 28:1–19. 23397514 10.1007/s10654-013-9762-6

[31] Wu R Wang T Gu R Xing D Ye C Chen Y. Hypertensive disorders of pregnancy and risk of cardiovascular disease-related morbidity and mortality: a systematic review and meta-analysis. *Cardiology* 2020; 145:633–647. 32841945 10.1159/000508036

[32] Haug EB Horn J Markovitz AR Fraser A Klykken B Dalen H. Association of conventional cardiovascular risk factors with cardiovascular disease after hypertensive disorders of pregnancy: analysis of the Nord-Trøndelag Health Study. *JAMA Cardiol* 2019; 4:628–635. 31188397 10.1001/jamacardio.2019.1746 PMC6563586

[33] Melchiorre K Giorgione V Thilaganathan B. The placenta and preeclampsia: villain or victim? *Am J Obstet Gynecol* 2021; 226:S954–S962. 33771361 10.1016/j.ajog.2020.10.024

[34] Thilaganathan B Kalafat E. Cardiovascular System in Preeclampsia and Beyond. *Hypertension* 2019; 73:522–531. 30712425 10.1161/HYPERTENSIONAHA.118.11191 PMC6380450

[35] Khosla K Heimberger S Nieman KM Tung A Shahul S Staff AC Rana S. Long-term cardiovascular disease risk in women after hypertensive disorders of pregnancy: recent advances in hypertension. *Hypertension* 2021; 78:927–935. 34397272 10.1161/HYPERTENSIONAHA.121.16506 PMC8678921

[36] Parada-Niño L Castillo-León LF Morel A. Preeclampsia, natural history, genes, and miRNAs associated with the syndrome. *J Pregnancy* 2022; 2022:3851225. 35198246 10.1155/2022/3851225 PMC8860533

[37] McGinnis R Steinthorsdottir V Williams NO Thorleifsson G Shooter S Hjartardottir S. Variants in the fetal genome near FLT1 are associated with risk of preeclampsia. *Nat Genet* 2017; 49:1255–1260. 28628106 10.1038/ng.3895

[38] Johnson MP Brennecke SP East CE Göring HHH Kent JWJ Dyer TD. Genome-wide association scan identifies a risk locus for preeclampsia on 2q14, near the inhibin, beta B gene. *PLoS One* 2012; 7:e33666. 22432041 10.1371/journal.pone.0033666 PMC3303857

[39] Zhao L Bracken MB DeWan AT. Genome-wide association study of preeclampsia detects novel maternal single nucleotide polymorphisms and copy-number variants in subsets of the Hyperglycemia and Adverse Pregnancy Outcome (HAPO) study cohort. *Ann Hum Genet* 2013; 77:277–287. 23551011 10.1111/ahg.12021 PMC3740040

[40] Zhao L Triche EW Walsh KM Bracken MB Saftlas AF Hoh J Dewan AT. Genome-wide association study identifies a maternal copy-number deletion in PSG11 enriched among preeclampsia patients. *BMC Pregnancy Childbirth* 2012; 12:61. 22748001 10.1186/1471-2393-12-61 PMC3476390

[41] Smith CJ Saftlas AF Spracklen CN Triche EW Bjonnes A Keating B. Genetic risk score for essential hypertension and risk of preeclampsia. *Am J Hypertens* 2016; 29:17–24. 26002928 10.1093/ajh/hpv069 PMC4692983

[42] Spracklen CN Saftlas AF Triche EW Bjonnes A Keating B Saxena R. Genetic predisposition to dyslipidemia and risk of preeclampsia. *Am J Hypertens* 2015; 28:915–923. 25523295 10.1093/ajh/hpu242 PMC4542907

[43] Tita AT Szychowski JM Boggess K Dugoff L Sibai B Lawrence K. Chronic Hypertension and Pregnancy (CHAP) Trial Consortium. Treatment for mild chronic hypertension during pregnancy. *New Engl J Med* 2022; 386:1781–1792. 35363951 10.1056/NEJMoa2201295 PMC9575330

[44] Garovic VD Dechend R Easterling T Karumanchi SA Baird SM Magee LA. American Heart Association Council on Hypertension; Council on the Kidney in Cardiovascular Disease, Kidney in Heart Disease Science Committee; Council on Arteriosclerosis, Thrombosis and Vascular Biology; Council on Lifestyle and Cardiometabolic Health; Council on Peripheral Vascular Disease; and Stroke Council. Hypertension in pregnancy: diagnosis, blood pressure goals, and pharmacotherapy: a scientific statement from the American Heart Association. *Hypertension* 2022; 79:E21–E41. 34905954 10.1161/HYP.0000000000000208 PMC9031058

