# Supplementary material for: Associations of polygenic risk scores for preeclampsia and blood pressure with hypertensive disorders of pregnancy
Source: J Hypertens. 2022 Dec 16;41(3):380–7. doi: 10.1097/HJH.0000000000003336 (PMC9894151; doi:10.1097/HJH.0000000000003336)
Supplement: Supplemental Digital Content [file jhype-41-380-s001.pdf]

SUPPLEMENTAL MATERIAL

Associations of Polygenic Risk Scores for Preeclampsia and Blood Pressure with Hypertensive Disorders of Pregnancy

Jouko Nurkkala[a,b] MD; Anni Kauko[c] PhD; FinnGen; Hannele Laivuori[d,e,f,g] MD, PhD; Tanja Saarela[h] MD PhD; Jaakko Tyrmi[i] PhD; Felix Vaura[c] BA; Susan Cheng[j,k] MD, MPH; Natalie A. Bello[j] MD, MPH; Jenni Aittokallio[a,b] MD, PhD; Teemu Niiranen[c,l,m] MD, PhD

Affiliations:

a. Division of Perioperative Services, Intensive Care and Pain Medicine, Turku University Hospital, Turku, Finland
b. Department of Anesthesiology and Intensive Care, University of Turku, Turku, Finland
c. Department of Internal Medicine, University of Turku, Turku, Finland
d. Department of Obstetrics and Gynecology, Tampere University Hospital, Tampere, Finland
e. Center for Child, Adolescent, and Maternal Health Research, Faculty of Medicine and Health Technology, Tampere University, Tampere, Finland
f. Institute for Molecular Medicine Finland (FIMM), Helsinki Institute of Life Science, University of Helsinki, Helsinki, Finland
g. Medical and Clinical Genetics, University of Helsinki and Helsinki University Hospital, Helsinki, Finland
h. Department of Clinical Genetics, Kuopio University Hospital, Kuopio, Finland
i. Faculty of Medicine and Health Technology, University of Tampere, Tampere, Finland
j. Smidt Heart Institute, Cedars-Sinai Medical Center, Los Angeles, CA, USA
k. Division of Cardiology Brigham and Women's Hospital, Boston, MA, USA
l. Division of Medicine, Turku University Hospital, Turku, Finland
m. Department of Public Health Solutions, Finnish Institute for Health and Welfare, Turku, Finland

Supplemental Methods

Ethics statement

Patients and control subjects in FinnGen provided informed consent for biobank research, based on the Finnish Biobank Act. Alternatively, separate research cohorts, collected prior the Finnish Biobank Act came into effect (in September 2013) and start of FinnGen (August 2017), were collected based on study-specific consents and later transferred to the Finnish biobanks after approval by Fimea (Finnish Medicines Agency), the National Supervisory Authority for Welfare and Health. Recruitment protocols followed the biobank protocols approved by Fimea. The Coordinating Ethics Committee of the Hospital District of Helsinki and Uusimaa (HUS) statement number for the FinnGen study is Nr HUS/990/2017.

The FinnGen study is approved by Finnish Institute for Health and Welfare (permit numbers: THL/2031/6.02.00/2017, THL/1101/5.05.00/2017, THL/341/6.02.00/2018, THL/2222/6.02.00/2018, THL/283/6.02.00/2019, THL/1721/5.05.00/2019 and THL/1524/5.05.00/2020), Digital and population data service agency (permit numbers: VRK43431/2017-3, VRK/6909/2018-3, VRK/4415/2019-3), the Social Insurance Institution (permit numbers: KELA 58/522/2017, KELA 131/522/2018, KELA 70/522/2019, KELA 98/522/2019, KELA 134/522/2019, KELA 138/522/2019, KELA 2/522/2020, KELA 16/522/2020), Findata permit numbers THL/2364/14.02/2020, THL/4055/14.06.00/2020,,THL/3433/14.06.00/2020, THL/4432/14.06/2020, THL/5189/14.06/2020, THL/5894/14.06.00/2020, THL/6619/14.06.00/2020, THL/209/14.06.00/2021, THL/688/14.06.00/2021, THL/1284/14.06.00/2021, THL/1965/14.06.00/2021, THL/5546/14.02.00/2020 and Statistics Finland (permit numbers: TK-53-1041-17 and TK/143/07.03.00/2020 (earlier TK-53-90-20)).

The Biobank Access Decisions for FinnGen samples and data utilized in FinnGen Data Freeze 7 include: THL Biobank BB2017_55, BB2017_111, BB2018_19, BB_2018_34, BB_2018_67, BB2018_71, BB2019_7, BB2019_8, BB2019_26, BB2020_1, Finnish Red Cross Blood Service Biobank 7.12.2017, Helsinki Biobank HUS/359/2017, Auria Biobank AB17-5154 and amendment #1 (August 17 2020), Biobank Borealis of Northern Finland_2017_1013, Biobank of Eastern Finland 1186/2018 and amendment 22 § /2020, Finnish Clinical Biobank Tampere MH0004 and amendments (21.02.2020 & 06.10.2020), Central Finland Biobank 1-2017, and Terveystalo Biobank STB 2018001.

Supplemental Table S1. Disease definitions.

Endpoint	FinnGen code	Hospital discharge and death registries	Drug reimbursement and purchase registries	Birth and population registries
Birth				BIRTH_YEAR ≥ 1969
Gestational hypertension	O15_GESTAT_HYPERT	ICD-10: O13; ICD-9: 6423; ICD-8: 63701		
Preeclampsia	O15_PREECLAMPS	ICD-10: O14; ICD-9: 642[4-5]; ICD-8: 6370[349]		
Multiple gestation				NRO_FETUSES ≥ 2
In vitro fertilization				IVF==1 or ICSI==1
Hypertension	I9_HYPTENS	ICD-10: I1[0-3]|I15|I674;<br><br>ICD-9: 4019X|4029A|4029B|4039A|4040A|4059A|4059B|4372A|4059X; ICD-8: 40[0-4]	DR: 205	
Diabetes	E4_DIABETES	E4_DM1 or E4_DM2 or E4_DMNAS or GEST_DIABETES		
	E4_DM1	ICD-10: E10; ICD-9: 250[0-8]B	ICD-10: E10	
	E4_DM2	ICD-10: E11; ICD-9: 250[0-8]A	ICD-10: E11; ATC: A10B	
	E4_DMNAS	ICD-10: E12; ICD-9: 250[0-8]C|250[0-8]X;<br><br>ICD-8: 2500[0-6]|2500[8-9]|250	ICD-10: E1[2-4]	
	GEST_DIABETES	ICD-10:O24.4; ICD-9: 6488A		
Renal failure	N14_RENFAIL	ICD-10: N17-N19|Y84.1|Z99.2;<br><br>ICD-9: 58[4-5]	DR: 13[7-8]	
Obesity	E4_OBESITY	ICD-10: E66; ICD-9: 2780|2788A; ICD-8: 27799		

Disease definitions in the Hospital Discharge, Cause of Death, Drug reimbursement and Drug purchase registries. The FinnGen codes are available at https://risteys.finngen.fi/ for more information. Diseases are defined by ICD-codes and Finnish Social Insurance Institution drug codes (DR: Drug reimbursement code, ATC: Anatomical therapeutic chemical code).

Table S2. Association between various models and preeclampsia.

Model	Odds Ratio (95% CI)	p value
Model 1		
BP-PRS	1.27 (1.24-1.30)	$9.6x10^{-80}$
Model 2		
PE-PRS	1.22 (1.19-1.25)	$2.6x10^{-57}$
Model 3		
BP-PRS	1.28 (1.24-1.31)	$2.0x10^{-80}$
Index age	1.06 (1.05-1.06)	$3.5x10^{-131}$
Multifetal pregnancy	3.35 (2.93-3.81)	$2.5x10^{-72}$
IVF	0.64 (0.32-1.15)	0.17
Prior Hypertension	4.21 (3.49-5.06)	$3.7x10^{-52}$
Prior Obesity	1.76 (1.46-2.10)	$1.0x10^{-9}$
Prior Diabetes	2.65 (2.44-2.87)	$4.9x10^{-124}$
Prior Renal Failure	1.27 (0.52-2.79)	0.57
Model 4		
PE-PRS	1.22 (1.19-1.25)	$6.0x10^{-56}$
Index age	1.06 (1.05-1.06)	$1.8x10^{-126}$
Multifetal pregnancy	3.32 (2.91-3.78)	$1.7x10^{-71}$
IVF	0.66 (0.33-1.17)	0.18
Prior Hypertension	4.53 (3.76-5.44)	$1.8x10^{-57}$
Prior Obesity	1.74 (1.45-2.08)	$1.6x10^{-9}$
Prior Diabetes	2.64 (2.44-2.86)	$1.3x10^{-123}$
Prior Renal Failure	1.17 (0.48-2.59)	0.71

CI, confidence interval; BP, Blood Pressure; PRS, polygenic risk score; Index age is the age in preeclamptic pregnancy; IVF, in vitro fertilization.

FinnGen

Full Name	Affiliation	E-mail	Role 1	Role 2
Aarno Palotie	Institute for Molecular Medicine, Finland (FIMM), HiLIFE, University of Helsinki, Helsinki, Finland; Broad Institute of MIT and Harvard; Massachusetts General Hospital	aarno.palotie@helsinki.fi	Steering Committee	Steering Committee
Mark Daly	Institute for Molecular Medicine, Finland (FIMM), HiLIFE, University of Helsinki, Helsinki, Finland; Broad Institute of MIT and Harvard; Massachusetts General Hospital	mark.daly@helsinki.fi	Steering Committee	Steering Committee
Bridget Riley-Gills	Abbvie, Chicago, IL, United States	bridget.rileygillis@abbvie.com	Steering Committee	Pharmaceutical companies
Howard Jacob	Abbvie, Chicago, IL, United States	howard.jacob@abbvie.com	Steering Committee	Pharmaceutical companies
Dirk Paul	Astra Zeneca, Cambridge, United Kingdom	dirk.paul@astrazeneca.com	Steering Committee	Pharmaceutical companies
Athena Matakidou	Astra Zeneca, Cambridge, United Kingdom	athena.x.matakidou@gsk.com	Steering Committee	Pharmaceutical companies
Adam Platt	Astra Zeneca, Cambridge, United Kingdom	adam.platt@astrazeneca.com	Steering Committee	Pharmaceutical companies
Heiko Runz	Biogen, Cambridge, MA, United States	heiko.runz@biogen.com	Steering Committee	Pharmaceutical companies
Sally John	Biogen, Cambridge, MA, United States	sally.john@biogen.com	Steering Committee	Pharmaceutical companies
George Okafo	Boehringer Ingelheim, Ingelheim am Rhein, Germany	george.okafo@boehringer-ingelheim.com	Steering Committee	Pharmaceutical companies
Nathan Lawless	Boehringer Ingelheim, Ingelheim am Rhein, Germany	nathan.lawless@boehringer-ingelheim.com	Steering Committee	Pharmaceutical companies
Robert Plenge	Bristol Myers Squibb, New York, NY, United States	robert.plenge@bms.com	Steering Committee	Pharmaceutical companies
Joseph Maranville	Bristol Myers Squibb, New York, NY, United States	joseph.maranville@bms.com	Steering Committee	Pharmaceutical companies
Mark McCarthy	Genentech, San Francisco, CA, United States	mccarthy.mark@gene.com	Steering Committee	Pharmaceutical companies
Julie Hunkapiller	Genentech, San Francisco, CA, United States	hunkapiller.julie@gene.com	Steering Committee	Pharmaceutical companies
Margaret G. Ehm	GlaxoSmithKline, Collegeville, PA, United States	meg.g.ehm@gsk.com	Steering Committee	Pharmaceutical companies
Kirsi Auro	GlaxoSmithKline, Espoo, Finland	kirsi.m.auro@gsk.com	Steering Committee	Pharmaceutical companies
Simonne Longerich	Merck, Kenilworth, NJ, United States	simonne.longerich@merck.com	Steering Committee	Pharmaceutical companies
Caroline Fox	Merck, Kenilworth, NJ, United States	caroline.fox@merck.com	Steering Committee	Pharmaceutical companies
Anders Mälarstig	Pfizer, New York, NY, United States	anders.malarstig@pfizer.com	Steering Committee	Pharmaceutical companies
Katherine Klinger	Translational Sciences, Sanofi R&D, Framingham, MA, USA	katherine.klinger@sanofi.com	Steering Committee	Pharmaceutical companies
Deepak Raipal	Translational Sciences, Sanofi R&D, Framingham, MA, USA	deepak.rajpal@sanofi.com	Steering Committee	Pharmaceutical companies
Eric Green	Maze Therapeutics, San Francisco, CA, United States	egreen@mazetx.com	Steering Committee	Pharmaceutical companies
Robert Graham	Maze Therapeutics, San Francisco, CA, United States	rgraham@mazetx.com	Steering Committee	Pharmaceutical companies
Robert Yang	Janssen Biotech, Beerse, Belgium	ryang31@its.jnj.com	Steering Committee	Pharmaceutical companies
Chris O´Donnell	Novartis Institutes for BioMedical Research, Cambridge, MA, United States	chris.odonnell@novartis.com	Steering Committee	Pharmaceutical companies
Tomi Mäkelä	HiLIFE, University of Helsinki, Finland, Finland	tomi.makela@helsinki.fi	Steering Committee	University of Helsinki & Biobanks
Jaakko Kaprio	Institute for Molecular Medicine Finland, HiLIFE, Helsinki, Finland, Finland	jaakko.kaprio@helsinki.fi	Steering Committee	University of Helsinki & Biobanks
Petri Virolainen	Auria Biobank / University of Turku / Hospital District of Southwest Finland, Turku, Finland	petri.virolainen@tyks.fi	Steering Committee	University of Helsinki & Biobanks
Antti Hakanen	Auria Biobank / University of Turku / Hospital District of Southwest Finland, Turku, Finland	antti.hakanen@tyks.fi	Steering Committee	University of Helsinki & Biobanks
Terhi Kilpi	THL Biobank / Finnish Institute for Health and Welfare (THL), Helsinki, Finland	terhi.kilpi@thl.fi	Steering Committee	University of Helsinki & Biobanks
Markus Perola	THL Biobank / Finnish Institute for Health and Welfare (THL), Helsinki, Finland	markus.perola@thl.fi	Steering Committee	University of Helsinki & Biobanks
Jukka Partanen	Finnish Red Cross Blood Service / Finnish Hematology Registry and Clinical Biobank, Helsinki, Finland	jukka.partanen@veripalvelu.fi	Steering Committee	University of Helsinki & Biobanks
Anne Pitkäranta	Helsinki Biobank / Helsinki University and Hospital District of Helsinki and Uusimaa, Helsinki	anne.pitkaranta@hus.fi	Steering Committee	University of Helsinki & Biobanks
Juhani Junttila	Northern Finland Biobank Borealis / University of Oulu / Northern Ostrobothnia Hospital District, Oulu, Finland	juhani.junttila@ppshp.fi	Steering Committee	University of Helsinki & Biobanks
Raisa Serpi	Northern Finland Biobank Borealis / University of Oulu / Northern Ostrobothnia Hospital District, Oulu, Finland	raisa.serpi@ppshp.fi	Steering Committee	University of Helsinki & Biobanks
Tarja Laitinen	Finnish Clinical Biobank Tampere / University of Tampere / Pirkanmaa Hospital District, Tampere, Finland	tarja.laitinen@pshp.fi	Steering Committee	University of Helsinki & Biobanks
Veli-Matti Kosma	Biobank of Eastern Finland / University of Eastern Finland / Northern Savo Hospital District, Kuopio, Finland	veli-matti.kosma@uef.fi	Steering Committee	University of Helsinki & Biobanks
Jari Laukkanen	Central Finland Biobank / University of Jyväskylä / Central Finland Health Care District, Jyväskylä, Finland	jari.laukkanen@ksshp.fi	Steering Committee	University of Helsinki & Biobanks
Marco Hautalahti	FINBB - Finnish biobank cooperative	marco.hautalahti@finbb.fi	Steering Committee	University of Helsinki & Biobanks
Outi Tuovila	Business Finland, Helsinki, Finland	outi.tuovila@businessfinland.fi	Steering Committee	Other Experts/ Non-Voting Members
Raimo Pakkanen	Business Finland, Helsinki, Finland	raimo.pakkanen@businessfinland.fi	Steering Committee	Other Experts/ Non-Voting Members
Jeffrey Waring	Abbvie, Chicago, IL, United States	jeff.waring@abbvie.com	Scientific Committee	Pharmaceutical companies
Bridget Riley-Gillis	Abbvie, Chicago, IL, United States	bridget.rileygillis@abbvie.com	Scientific Committee	Pharmaceutical companies
Fedik Rahimov	Abbvie, Chicago, IL, United States	fedik.rahimov@abbvie.com	Scientific Committee	Pharmaceutical companies
Ioanna Tachmazidou	Astra Zeneca, Cambridge, United Kingdom	ioanna.tachmazidou@astrazeneca.com	Scientific Committee	Pharmaceutical companies
Chia-Yen Chen	Biogen, Cambridge, MA, United States	chiayen.chen@biogen.com	Scientific Committee	Pharmaceutical companies
Heiko Runz	Biogen, Cambridge, MA, United States	heiko.runz@biogen.com	Scientific Committee	Pharmaceutical companies
Zhihao Ding	Boehringer Ingelheim, Ingelheim am Rhein, Germany	zhihao.ding@boehringer-ingelheim.com	Scientific Committee	Pharmaceutical companies
Marc Jung	Boehringer Ingelheim, Ingelheim am Rhein, Germany	marc_oliver.jung@boehringer-ingelheim.com	Scientific Committee	Pharmaceutical companies
Shameek Biswas	Bristol Myers Squibb, New York, NY, United States	Shameek.Biswas@bms.com	Scientific Committee	Pharmaceutical companies
Rion Pendergrass	Genentech, San Francisco, CA, United States	penders2@gene.com	Scientific Committee	Pharmaceutical companies
Julie Hunkapiller	Genentech, San Francisco, CA, United States	hunkapiller.julie@gene.com	Scientific Committee	Pharmaceutical companies
Margaret G. Ehm	GlaxoSmithKline, Collegeville, PA, United States	meg.g.ehm@gsk.com	Scientific Committee	Pharmaceutical companies
David Pulford	GlaxoSmithKline, Stevenage, United Kingdom	david.x.pulford@gsk.com	Scientific Committee	Pharmaceutical companies
Neha Raghavan	Merck, Kenilworth, NJ, United States	neha.raghavan@merck.com	Scientific Committee	Pharmaceutical companies
Adriana Huertas-Vazquez	Merck, Kenilworth, NJ, United States	adriana.huertas.vazquez@merck.com	Scientific Committee	Pharmaceutical companies
Jae-Hoon Sul	Merck, Kenilworth, NJ, United States	jae.hoon.sul@merck.com	Scientific Committee	Pharmaceutical companies
Anders Mälarstig	Pfizer, New York, NY, United States	anders.malarstig@pfizer.com	Scientific Committee	Pharmaceutical companies
Xinli Hu	Pfizer, New York, NY, United States	xinli.hu@pfizer.com	Scientific Committee	Pharmaceutical companies
Katherine Klinger	Translational Sciences, Sanofi R&D, Framingham, MA, USA	katherine.klinger@sanofi.com	Scientific Committee	Pharmaceutical companies
Robert Graham	Maze Therapeutics, San Francisco, CA, United States	rgraham@mazetx.com	Scientific Committee	Pharmaceutical companies
Eric Green	Maze Therapeutics, San Francisco, CA, United States	egreen@mazetx.com	Scientific Committee	Pharmaceutical companies
Sahar Mozaffari	Maze Therapeutics, San Francisco, CA, United States	smozaffari@mazetx.com	Scientific Committee	Pharmaceutical companies
Dawn Waterworth	Janssen Research & Development, LLC, Spring House, PA, United States	dwaterwo@its.jnj.com	Scientific Committee	Pharmaceutical companies
Nicole Renaud	Novartis Institutes for BioMedical Research, Cambridge, MA, United States	nicole.renaud@novartis.com	Scientific Committee	Pharmaceutical companies
Ma´en Obeidat	Novartis Institutes for BioMedical Research, Cambridge, MA, United States	maen.obeidat@novartis.com	Scientific Committee	Pharmaceutical companies
Samuli Ripatti	Institute for Molecular Medicine Finland, HiLIFE, Helsinki, Finland	samuli.ripatti@helsinki.fi	Scientific Committee	University of Helsinki & Biobanks
Johanna Schleutker	Auria Biobank / Univ. of Turku / Hospital District of Southwest Finland, Turku, Finland	johanna.schleutker@utu.fi	Scientific Committee	University of Helsinki & Biobanks
Markus Perola	THL Biobank / Finnish Institute for Health and Welfare (THL), Helsinki, Finland	markus.perola@thl.fi	Scientific Committee	University of Helsinki & Biobanks
Mikko Arvas	Finnish Red Cross Blood Service / Finnish Hematology Registry and Clinical Biobank, Helsinki, Finland	mikko.arvas@veripalvelu.fi	Scientific Committee	University of Helsinki & Biobanks
Olli Carpén	Helsinki Biobank / Helsinki University and Hospital District of Helsinki and Uusimaa, Helsinki	olli.carpen@helsinki.fi	Scientific Committee	University of Helsinki & Biobanks
Reetta Hinttala	Northern Finland Biobank Borealis / University of Oulu / Northern Ostrobothnia Hospital District, Oulu, Finland	reetta.hinttala@oulu.fi	Scientific Committee	University of Helsinki & Biobanks
Johannes Kettunen	Northern Finland Biobank Borealis / University of Oulu / Northern Ostrobothnia Hospital District, Oulu, Finland	johannes.kettunen@oulu.fi	Scientific Committee	University of Helsinki & Biobanks
Arto Mannermaa	Biobank of Eastern Finland / University of Eastern Finland / Northern Savo Hospital District, Kuopio, Finland	arto.mannermaa@uef.fi	Scientific Committee	University of Helsinki & Biobanks
Katriina Aalto-Setälä	Faculty of Medicine and Health Technology, Tampere University, Tampere, Finland	katriina.aalto-setala@tuni.fi	Scientific Committee	University of Helsinki & Biobanks
Mika Kähönen	Finnish Clinical Biobank Tampere / University of Tampere / Pirkanmaa Hospital District, Tampere, Finland	mika.kahonen@uta.fi	Scientific Committee	University of Helsinki & Biobanks
Jari Laukkanen	Central Finland Biobank / University of Jyväskylä / Central Finland Health Care District, Jyväskylä, Finland	jari.laukkanen@ksshp.fi	Scientific Committee	University of Helsinki & Biobanks
Johanna Mäkelä	FINBB - Finnish biobank cooperative	johanna.makela@finbb.fi	Scientific Committee	University of Helsinki & Biobanks
Reetta Kälviäinen	Northern Savo Hospital District, Kuopio, Finland	reetta.kalviainen@kuh.fi	Clinical Groups	Neurology Group
Valtteri Julkunen	Northern Savo Hospital District, Kuopio, Finland	valtteri.julkunen@kuh.fi	Clinical Groups	Neurology Group
Hilkka Soininen	Northern Savo Hospital District, Kuopio, Finland	hilkka.soininen@uef.fi	Clinical Groups	Neurology Group
Anne Remes	Northern Ostrobothnia Hospital District, Oulu, Finland	anne.remes@oulu.fi	Clinical Groups	Neurology Group
Mikko Hiltunen	University of Eastern Finland, Kuopio, Finland	mikko.hiltunen@uef.fi	Clinical Groups	Neurology Group
Jukka Peltola	Pirkanmaa Hospital District, Tampere, Finland	jukka.peltola@pshp.fi	Clinical Groups	Neurology Group
Minna Raivio	Hospital District of Helsinki and Uusimaa, Helsinki, Finland	minna.raivio@geri.fi	Clinical Groups	Neurology Group
Pentti Tienari	Hospital District of Helsinki and Uusimaa, Helsinki, Finland	pentti.tienari@hus.fi	Clinical Groups	Neurology Group
Juha Rinne	Hospital District of Southwest Finland, Turku, Finland	juha.rinne@tyks.fi	Clinical Groups	Neurology Group
Roosa Kallionpää	Hospital District of Southwest Finland, Turku, Finland	roosa.kallionpaa@tyks.fi	Clinical Groups	Neurology Group
Juulia Partanen	Institute for Molecular Medicine Finland, HiLIFE, University of Helsinki, Finland	juulia.partanen@helsinki.fi	Clinical Groups	Neurology Group
Ali Abbasi	Abbvie, Chicago, IL, United States	ali.abbasi@abbvie.com	Clinical Groups	Neurology Group
Adam Ziemann	Abbvie, Chicago, IL, United States	adam.ziemann@abbvie.com	Clinical Groups	Neurology Group
Nizar Smaoui	Abbvie, Chicago, IL, United States	nizar.smaoui@abbvie.com	Clinical Groups	Neurology Group

Anne Lehtonen	Abbvie, Chicago, IL, United States	anne.lehtonen@abbvie.com	Clinical Groups	Neurology Group
Susan Eaton	Biogen, Cambridge, MA, United States	susan.eaton@biogen.com	Clinical Groups	Neurology Group
Heiko Runz	Biogen, Cambridge, MA, United States	heiko.runz@biogen.com	Clinical Groups	Neurology Group
Sanni Lahdenperä	Biogen, Cambridge, MA, United States	sanni.lahdenpera@biogen.com	Clinical Groups	Neurology Group
Shameek Biswas	Bristol Myers Squibb, New York, NY, United States	shameek.biswas@bms.com	Clinical Groups	Neurology Group
Julie Hunkapiller	Genentech, San Francisco, CA, United States	hunkapiller.julie@gene.com	Clinical Groups	Neurology Group
Natalie Bowers	Genentech, San Francisco, CA, United States	bowersn1@gene.com	Clinical Groups	Neurology Group
Edmond Teng	Genentech, San Francisco, CA, United States	teng.edmond@gene.com	Clinical Groups	Neurology Group
Rion Pendergrass	Genentech, San Francisco, CA, United States	penders2@gene.com	Clinical Groups	Neurology Group
Fanli Xu	GlaxoSmithKline, Brentford, United Kingdom	chun-fang.2.xu@gsk.com	Clinical Groups	Neurology Group
David Pulford	GlaxoSmithKline, Stevenage, United Kingdom	david.x.pulford@gsk.com	Clinical Groups	Neurology Group
Kirsi Auro	GlaxoSmithKline, Espoo, Finland	kirsi.m.auro@gsk.com	Clinical Groups	Neurology Group
Laura Addis	GlaxoSmithKline, Brentford, United Kingdom	laura.x.addis@gsk.com	Clinical Groups	Neurology Group
John Eicher	GlaxoSmithKline, Brentford, United Kingdom	john.d.eicher@gsk.com	Clinical Groups	Neurology Group
Qingqin S Li	Janssen Research & Development, LLC, Titusville, NJ 08560, United States	QLi2@its.jnj.com	Clinical Groups	Neurology Group
Karen He	Janssen Research & Development, LLC, Spring House, PA, United States	khe2@its.jnj.com	Clinical Groups	Neurology Group
Ekaterina Khramtsova	Janssen Research & Development, LLC, Spring House, PA, United States	ekhramts@its.jnj.com	Clinical Groups	Neurology Group
Neha Raghavan	Merck, Kenilworth, NJ, United States	neha.raghavan@merck.com	Clinical Groups	Neurology Group
Martti Färkkilä	Hospital District of Helsinki and Uusimaa, Helsinki, Finland	martti.farkkila@hus.fi	Clinical Groups	Gastroenterology Group
Jukka Koskela	Hospital District of Helsinki and Uusimaa, Helsinki, Finland	jukka.koskela@helsinki.fi	Clinical Groups	Gastroenterology Group
Sampsa Pikkarainen	Hospital District of Helsinki and Uusimaa, Helsinki, Finland	sampsa.pikkarainen@hus.fi	Clinical Groups	Gastroenterology Group
Airi Jussila	Pirkanmaa Hospital District, Tampere, Finland	airi.jussila@pshp.fi	Clinical Groups	Gastroenterology Group
Katri Kaukinen	Pirkanmaa Hospital District, Tampere, Finland	katri.kaukinen@tuni.fi	Clinical Groups	Gastroenterology Group
Timo Blomster	Northern Ostrobothnia Hospital District, Oulu, Finland	timo.blomster@ppshp.fi	Clinical Groups	Gastroenterology Group
Mikko Kiviniemi	Northern Savo Hospital District, Kuopio, Finland	mikko.kiviniemi@kuh.fi	Clinical Groups	Gastroenterology Group
Markku Voutilainen	Hospital District of Southwest Finland, Turku, Finland	markku.voutilainen@tyks.fi	Clinical Groups	Gastroenterology Group
Mark Daly	Institute for Molecular Medicine, Finland (FIMM), HiLIFE, University of Helsinki, Helsinki, Finland; Broad Institute of MIT and Harvard; Massachusetts General Hospital	mark.daly@helsinki.fi	Clinical Groups	Gastroenterology Group
Ali Abbasi	Abbvie, Chicago, IL, United States	ali.abbasi@abbvie.com	Clinical Groups	Gastroenterology Group
Jeffrey Waring	Abbvie, Chicago, IL, United States	jeff.waring@abbvie.com	Clinical Groups	Gastroenterology Group
Nizar Smaoui	Abbvie, Chicago, IL, United States	nizar.smaoui@abbvie.com	Clinical Groups	Gastroenterology Group
Fedik Rahimov	Abbvie, Chicago, IL, United States	fedik.rahimov@abbvie.com	Clinical Groups	Gastroenterology Group
Anne Lehtonen	Abbvie, Chicago, IL, United States	anne.lehtonen@abbvie.com	Clinical Groups	Gastroenterology Group
Tim Lu	Genentech, San Francisco, CA, United States	lut8@gene.com	Clinical Groups	Gastroenterology Group
Natalie Bowers	Genentech, San Francisco, CA, United States	bowersn1@gene.com	Clinical Groups	Gastroenterology Group
Rion Pendergrass	Genentech, San Francisco, CA, United States	penders2@gene.com	Clinical Groups	Gastroenterology Group
Linda McCarthy	GlaxoSmithKline, Brentford, United Kingdom	linda.c.mccarthy@gsk.com	Clinical Groups	Gastroenterology Group
Amy Hart	Janssen Research & Development, LLC, Spring House, PA, United States	ahart13@its.jnj.com	Clinical Groups	Gastroenterology Group
Meijian Guan	Janssen Research & Development, LLC, Spring House, PA, United States	mguan4@its.jnj.com	Clinical Groups	Gastroenterology Group
Jason Miller	Merck, Kenilworth, NJ, United States	jason.miller4@merck.com	Clinical Groups	Gastroenterology Group
Kirsi Kalpala	Pfizer, New York, NY, United States	kirsi.kalpala@pfizer.com	Clinical Groups	Gastroenterology Group
Melissa Miller	Pfizer, New York, NY, United States	melissa.r.miller@pfizer.com	Clinical Groups	Gastroenterology Group
Xinli Hu	Pfizer, New York, NY, United States	xinli.hu@pfizer.com	Clinical Groups	Gastroenterology Group
Kari Eklund	Hospital District of Helsinki and Uusimaa, Helsinki, Finland	kari.eklund@hus.fi	Clinical Groups	Rheumatology Group
Antti Palomäki	Hospital District of Southwest Finland, Turku, Finland	ajpalo@utu.fi	Clinical Groups	Rheumatology Group
Pia Isomäki	Pirkanmaa Hospital District, Tampere, Finland	pia.isomaki@pshp.fi	Clinical Groups	Rheumatology Group
Laura Pirilä	Hospital District of Southwest Finland, Turku, Finland	laura.pirila@fimnet.fi,laura.pirila@tyks.fi	Clinical Groups	Rheumatology Group
Oili Kaipiainen-Seppänen	Northern Savo Hospital District, Kuopio, Finland	oili.kaipiainen-seppanen@kuh.fi	Clinical Groups	Rheumatology Group
Johanna Huhtakangas	Northern Ostrobothnia Hospital District, Oulu, Finland	johanna.huhtakangas@kuh.fi	Clinical Groups	Rheumatology Group
Nina Mars	Institute for Molecular Medicine Finland, HiLIFE, Helsinki, Finland	nina.mars@helsinki.fi	Clinical Groups	Rheumatology Group
Ali Abbasi	Abbvie, Chicago, IL, United States	ali.abbasi@abbvie.com	Clinical Groups	Rheumatology Group
Jeffrey Waring	Abbvie, Chicago, IL, United States	jeff.waring@abbvie.com	Clinical Groups	Rheumatology Group
Fedik Rahimov	Abbvie, Chicago, IL, United States	fedik.rahimov@abbvie.com	Clinical Groups	Rheumatology Group
Apinya Lertratanakul	Abbvie, Chicago, IL, United States	apinya.lertratanakul@abbvie.com	Clinical Groups	Rheumatology Group
Nizar Smaoui	Abbvie, Chicago, IL, United States	nizar.smaoui@abbvie.com	Clinical Groups	Rheumatology Group
Anne Lehtonen	Abbvie, Chicago, IL, United States	anne.lehtonen@abbvie.com	Clinical Groups	Rheumatology Group
David Close	Astra Zeneca, Cambridge, United Kingdom	david.close@astrazeneca.com	Clinical Groups	Rheumatology Group
Marla Hochfeld	Bristol Myers Squibb, New York, NY, United States	mhochfeld@celgene.com	Clinical Groups	Rheumatology Group
Natalie Bowers	Genentech, San Francisco, CA, United States	bowersn1@gene.com	Clinical Groups	Rheumatology Group
Rion Pendergrass	Genentech, San Francisco, CA, United States	penders2@gene.com	Clinical Groups	Rheumatology Group
Jorge Esparza Gordillo	GlaxoSmithKline, Brentford, United Kingdom	jorge.x.esparza-gordillo@gsk.com	Clinical Groups	Rheumatology Group
Kirsi Auro	GlaxoSmithKline, Espoo, Finland	kirsi.m.auro@gsk.com	Clinical Groups	Rheumatology Group
Dawn Waterworth	Janssen Research & Development, LLC, Spring House, PA, United States	dwaterwo@its.jnj.com	Clinical Groups	Rheumatology Group
Fabiana Farias	Merck, Kenilworth, NJ, United States	fabiana.farias@merck.com	Clinical Groups	Rheumatology Group
Kirsi Kalpala	Pfizer, New York, NY, United States	kirsi.kalpala@pfizer.com	Clinical Groups	Rheumatology Group
Nan Bing	Pfizer, New York, NY, United States	nan.bing@pfizer.com	Clinical Groups	Rheumatology Group
Xinli Hu	Pfizer, New York, NY, United States	xinli.hu@pfizer.com	Clinical Groups	Rheumatology Group
Tarja Laitinen	Pirkanmaa Hospital District, Tampere, Finland	tarja.laitinen@pshp.fi	Clinical Groups	Pulmonology Group
Margit Pelkonen	Northern Savo Hospital District, Kuopio, Finland	margit.pelkonen@kuh.fi	Clinical Groups	Pulmonology Group
Paula Kauppi	Hospital District of Helsinki and Uusimaa, Helsinki, Finland	paula.kauppi@hus.fi	Clinical Groups	Pulmonology Group
Hannu Kankaanranta	University of Gothenburg, Gothenburg, Sweden/ Seinäjoki Central Hospital, Seinäjoki, Finland/ Tampere University, Tampere, Finland	hannu.kankaanranta@tuni.fi	Clinical Groups	Pulmonology Group
Terttu Harju	Northern Ostrobothnia Hospital District, Oulu, Finland	terttu.harju@oulu.fi	Clinical Groups	Pulmonology Group
Riitta Lahesmaa	Hospital District of Southwest Finland, Turku, Finland	rilahes@utu.fi	Clinical Groups	Pulmonology Group
Nizar Smaoui	Abbvie, Chicago, IL, United States	nizar.smaoui@abbvie.com	Clinical Groups	Pulmonology Group
Alex Mackay	Astra Zeneca, Cambridge, United Kingdom	alex.mackay@astrazeneca.com	Clinical Groups	Pulmonology Group
Glenda Lassi	Astra Zeneca, Cambridge, United Kingdom	glenda.lassi@astrazeneca.com	Clinical Groups	Pulmonology Group
Susan Eaton	Biogen, Cambridge, MA, United States	susan.eaton@biogen.com	Clinical Groups	Pulmonology Group
Hubert Chen	Genentech, San Francisco, CA, United States	chenh37@gene.com	Clinical Groups	Pulmonology Group
Rion Pendergrass	Genentech, San Francisco, CA, United States	penders2@gene.com	Clinical Groups	Pulmonology Group
Natalie Bowers	Genentech, San Francisco, CA, United States	bowersn1@gene.com	Clinical Groups	Pulmonology Group
Joanna Betts	GlaxoSmithKline, Brentford, United Kingdom	joanna.c.betts@gsk.com	Clinical Groups	Pulmonology Group
Kirsi Auro	GlaxoSmithKline, Espoo, Finland	kirsi.m.auro@gsk.com	Clinical Groups	Pulmonology Group
Rajashree Mishra	GlaxoSmithKline, Brentford, United Kingdom	rajashree.x.mishra@gsk.com	Clinical Groups	Pulmonology Group
Majd Mouded	Novartis, Basel, Switzerland	majd.mouded@novartis.com	Clinical Groups	Pulmonology Group
Debby Ngo	Novartis, Basel, Switzerland	debby.ngo@novartis.com	Clinical Groups	Pulmonology Group
Teemu Niiranen	Finnish Institute for Health and Welfare (THL), Helsinki, Finland	teemu.niiranen@thl.fi	Clinical Groups	Cardiometabolic Diseases Group
Felix Vaura	Finnish Institute for Health and Welfare (THL), Helsinki, Finland	fechva@utu.fi	Clinical Groups	Cardiometabolic Diseases Group
Veikko Salomaa	Finnish Institute for Health and Welfare (THL), Helsinki, Finland	veikko.salomaa@thl.fi	Clinical Groups	Cardiometabolic Diseases Group
Kaj Metsärinne	Hospital District of Southwest Finland, Turku, Finland	kaj.metsarinne@tyks.fi	Clinical Groups	Cardiometabolic Diseases Group
Jenni Aittokallio	Hospital District of Southwest Finland, Turku, Finland	jemato@utu.fi	Clinical Groups	Cardiometabolic Diseases Group
Mika Kähönen	Pirkanmaa Hospital District, Tampere, Finland	mika.kahonen@uta.fi	Clinical Groups	Cardiometabolic Diseases Group
Jussi Hernesniemi	Pirkanmaa Hospital District, Tampere, Finland	jussi.hernesniemi@tuni.fi	Clinical Groups	Cardiometabolic Diseases Group
Daniel Gordin	Hospital District of Helsinki and Uusimaa, Helsinki, Finland	daniel.gordin@hus.fi	Clinical Groups	Cardiometabolic Diseases Group
Juha Sinisalo	Hospital District of Helsinki and Uusimaa, Helsinki, Finland	juha.sinisalo@hus.fi	Clinical Groups	Cardiometabolic Diseases Group
Marja-Riitta Taskinen	Hospital District of Helsinki and Uusimaa, Helsinki, Finland	marja-riitta.taskinen@helsinki.fi	Clinical Groups	Cardiometabolic Diseases Group
Tiinamaija Tuomi	Hospital District of Helsinki and Uusimaa, Helsinki, Finland	tiinamaija.tuomi@hus.fi	Clinical Groups	Cardiometabolic Diseases Group
Timo Hiltunen	Hospital District of Helsinki and Uusimaa, Helsinki, Finland	timo.hiltunen@hus.fi	Clinical Groups	Cardiometabolic Diseases Group
Jari Laukkanen	Central Finland Health Care District, Jyväskylä, Finland	jari.laukkanen@ksshp.fi	Clinical Groups	Cardiometabolic Diseases Group
Amanda Elliott	Institute for Molecular Medicine Finland, HiLIFE, University of Helsinki, Finland; Broad Institute, Cambridge, MA, USA and Massachusetts General Hospital, Boston, MA, USA	aelliott@broadinstitute.org	Clinical Groups	Cardiometabolic Diseases Group
Mary Pat Reeve	Institute for Molecular Medicine Finland, HiLIFE, University of Helsinki, Finland	mary.reeve@helsinki.fi	Clinical Groups	Cardiometabolic Diseases Group
Sanni Ruotsalainen	Institute for Molecular Medicine Finland, HiLIFE, University of Helsinki, Finland	sanni.ruotsalainen@helsinki.fi	Clinical Groups	Cardiometabolic Diseases Group
Benjamin Challis	Astra Zeneca, Cambridge, United Kingdom	benjamin.challis@astrazeneca.com	Clinical Groups	Cardiometabolic Diseases Group
Dirk Paul	Astra Zeneca, Cambridge, United Kingdom	dirk.paul@astrazeneca.com	Clinical Groups	Cardiometabolic Diseases Group
Julie Hunkapiller	Genentech, San Francisco, CA, United States	hunkapiller.julie@gene.com	Clinical Groups	Cardiometabolic Diseases Group

Natalie Bowers	Genentech, San Francisco, CA, United States	bowersn1@gene.com	Clinical Groups	**Cardiometabolic Diseases Group**
Rion Pendergrass	Genentech, San Francisco, CA, United States	penders2@gene.com	Clinical Groups	**Cardiometabolic Diseases Group**
Audrey Chu	GlaxoSmithKline, Brentford, United Kingdom	audrey.y.chu@gsk.com	Clinical Groups	**Cardiometabolic Diseases Group**
Kirsi Auro	GlaxoSmithKline, Espoo, Finland	kirsi.m.auro@gsk.com	Clinical Groups	**Cardiometabolic Diseases Group**
Dermot Reilly	Janssen Research & Development, LLC, Boston, MA, United States	dreill11@its.jnj.com	Clinical Groups	**Cardiometabolic Diseases Group**
Mike Mendelson	Novartis, Boston, MA, United States	mike.mendelson@novartis.com	Clinical Groups	**Cardiometabolic Diseases Group**
Jaakko Parkkinen	Pfizer, New York, NY, United States	jaakko.parkkinen@pfizer.com	Clinical Groups	**Cardiometabolic Diseases Group**
Melissa Miller	Pfizer, New York, NY, United States	melissa.r.miller@pfizer.com	Clinical Groups	**Cardiometabolic Diseases Group**
Tuomo Meretoja	Hospital District of Helsinki and Uusimaa, Helsinki, Finland	tuomo.meretoja@hus.fi	Clinical Groups	**Oncology Group**
Heikki Joensuu	Hospital District of Helsinki and Uusimaa, Helsinki, Finland	heikki.joensuu@hus.fi	Clinical Groups	**Oncology Group**
Olli Carpén	Hospital District of Helsinki and Uusimaa, Helsinki, Finland	olli.carpen@helsinki.fi	Clinical Groups	**Oncology Group**
Johanna Mattson	Hospital District of Helsinki and Uusimaa, Helsinki, Finland	johanna.mattson@hus.fi	Clinical Groups	**Oncology Group**
Eveliina Salminen	Hospital District of Helsinki and Uusimaa, Helsinki, Finland	eveliina.e.salminen@hus.fi	Clinical Groups	**Oncology Group**
Annika Auranen	Pirkanmaa Hospital District , Tampere, Finland	anaura@utu.fi	Clinical Groups	**Oncology Group**
Peeter Karihtala	Northern Ostrobothnia Hospital District, Oulu, Finland	peeter.karihtala@oulu.fi	Clinical Groups	**Oncology Group**
Päivi Auvinen	Northern Savo Hospital District, Kuopio, Finland	paivi.auvinen@kuh.fi	Clinical Groups	**Oncology Group**
Klaus Elenius	Hospital District of Southwest Finland, Turku, Finland	klaus.elenius@utu.fi	Clinical Groups	**Oncology Group**
Johanna Schleutker	Hospital District of Southwest Finland, Turku, Finland	johanna.schleutker@utu.fi	Clinical Groups	**Oncology Group**
Esa Pitkänen	Institute for Molecular Medicine Finland, HiLIFE, University of Helsinki, Finland	esa.pitkanen@helsinki.fi	Clinical Groups	**Oncology Group**
Nina Mars	Institute for Molecular Medicine Finland, HiLIFE, University of Helsinki, Finland	nina.mars@helsinki.fi	Clinical Groups	**Oncology Group**
Mark Daly	Institute for Molecular Medicine, Finland (FIMM), HiLIFE, University of Helsinki, Helsinki, Finland; Broad Institute of MIT and Harvard; Massachusetts General Hospital	mark.daly@helsinki.fi	Clinical Groups	**Oncology Group**
Relja Popovic	Abbvie, Chicago, IL, United States	relja.popovic@abbvie.com	Clinical Groups	**Oncology Group**
Jeffrey Waring	Abbvie, Chicago, IL, United States	jeff.waring@abbvie.com	Clinical Groups	**Oncology Group**
Bridget Riley-Gillis	Abbvie, Chicago, IL, United States	bridget.rileygillis@abbvie.com	Clinical Groups	**Oncology Group**
Anne Lehtonen	Abbvie, Chicago, IL, United States	anne.lehtonen@abbvie.com	Clinical Groups	**Oncology Group**
Jennifer Schutzman	Genentech, San Francisco, CA, United States	schutzman.jennifer@gene.com	Clinical Groups	**Oncology Group**
Julie Hunkapiller	Genentech, San Francisco, CA, United States	hunkapiller.julie@gene.com	Clinical Groups	**Oncology Group**
Natalie Bowers	Genentech, San Francisco, CA, United States	bowersn1@gene.com	Clinical Groups	**Oncology Group**
Rion Pendergrass	Genentech, San Francisco, CA, United States	penders2@gene.com	Clinical Groups	**Oncology Group**
Diptee Kulkarni	GlaxoSmithKline, Brentford, United Kingdom	diptee.a.kulkarni@gsk.com	Clinical Groups	**Oncology Group**
Kirsi Auro	GlaxoSmithKline, Espoo, Finland	kirsi.m.auro@gsk.com	Clinical Groups	**Oncology Group**
Alessandro Porello	Janssen Research & Development, LLC, Spring House, PA, United States	APorrell@ITS.JNJ.com	Clinical Groups	**Oncology Group**
Andrey Loboda	Merck, Kenilworth, NJ, United States	andrey_loboda@merck.com	Clinical Groups	**Oncology Group**
Heli Lehtonen	Pfizer, New York, NY, United States	heli.lehtonen@pfizer.com	Clinical Groups	**Oncology Group**
Stefan McDonough	Pfizer, New York, NY, United States	stefan.McDonough@pfizer.com	Clinical Groups	**Oncology Group**
Sauli Vuoti	Janssen-Cilag Oy, Espoo, Finland	svuoti@its.jnj.com	Clinical Groups	**Oncology Group**
Kai Kaarniranta	Northern Savo Hospital District, Kuopio, Finland	kai.kaarniranta@uef.fi	Clinical Groups	**Opthalmology Group**
Joni A Turunen	Helsinki University Hospital and University of Helsinki, Helsinki, Finland; Eye Genetics Group, Folkhälsan Research Center, Helsinki, Finland	joni.turunen@helsinki.fi	Clinical Groups	**Opthalmology Group**
Terhi Ollila	Hospital District of Helsinki and Uusimaa, Helsinki, Finland	terhi.ollila@hus.fi	Clinical Groups	**Opthalmology Group**
Hannu Uusitalo	Pirkanmaa Hospital District, Tampere, Finland	hannu.uusitalo@tuni.fi	Clinical Groups	**Opthalmology Group**
Juha Karjalainen	Institute for Molecular Medicine Finland, HiLIFE, University of Helsinki, Finland	juha.karjalainen@helsinki.fi	Clinical Groups	**Opthalmology Group**
Esa Pitkänen	Institute for Molecular Medicine Finland, HiLIFE, University of Helsinki, Finland	esa.pitkanen@helsinki.fi	Clinical Groups	**Opthalmology Group**
Mengzhen Liu	Abbvie, Chicago, IL, United States	mengzhen.liu@abbvie.com	Clinical Groups	**Opthalmology Group**
Heiko Runz	Biogen, Cambridge, MA, United States	heiko.runz@biogen.com	Clinical Groups	**Opthalmology Group**
Stephanie Loomis	Biogen, Cambridge, MA, United States	stephanie.loomis@biogen.com	Clinical Groups	**Opthalmology Group**
Erich Strauss	Genentech, San Francisco, CA, United States	strauss.erich@gene.com	Clinical Groups	**Opthalmology Group**
Natalie Bowers	Genentech, San Francisco, CA, United States	bowersn1@gene.com	Clinical Groups	**Opthalmology Group**
Hao Chen	Genentech, San Francisco, CA, United States	haoc@gene.com	Clinical Groups	**Opthalmology Group**
Rion Pendergrass	Genentech, San Francisco, CA, United States	penders2@gene.com	Clinical Groups	**Opthalmology Group**
Kaisa Tasanen	Northern Ostrobothnia Hospital District, Oulu, Finland	kaisa.tasanen-maatta@oulu.fi	Clinical Groups	**Dermatology Group**
Laura Huilaja	Northern Ostrobothnia Hospital District, Oulu, Finland	laura.huilaja@oulu.fi	Clinical Groups	**Dermatology Group**
Katariina Hannula-Jouppi	Hospital District of Helsinki and Uusimaa, Helsinki, Finland	katariina.hannula-jouppi@hus.fi	Clinical Groups	**Dermatology Group**
Teea Salmi	Pirkanmaa Hospital District, Tampere, Finland	teea.salmi@pshp.fi	Clinical Groups	**Dermatology Group**
Sirkku Peltonen	Hospital District of Southwest Finland, Turku, Finland	sipelto@utu.fi	Clinical Groups	**Dermatology Group**
Leena Koulu	Hospital District of Southwest Finland, Turku, Finland	leena.koulu@tyks.fi	Clinical Groups	**Dermatology Group**
Nizar Smaoui	Abbvie, Chicago, IL, United States	nizar.smaoui@abbvie.com	Clinical Groups	**Dermatology Group**
Fedik Rahimov	Abbvie, Chicago, IL, United States	fedik.rahimov@abbvie.com	Clinical Groups	**Dermatology Group**
Anne Lehtonen	Abbvie, Chicago, IL, United States	anne.lehtonen@abbvie.com	Clinical Groups	**Dermatology Group**
David Choy	Genentech, San Francisco, CA, United States	choy.david@gene.com	Clinical Groups	**Dermatology Group**
Rion Pendergrass	Genentech, San Francisco, CA, United States	penders2@gene.com	Clinical Groups	**Dermatology Group**
Dawn Waterworth	Janssen Research & Development, LLC, Spring House, PA, United States	dwaterwo@its.jnj.com	Clinical Groups	**Dermatology Group**
Kirsi Kalpala	Pfizer, New York, NY, United States	kirsi.kalpala@pfizer.com	Clinical Groups	**Dermatology Group**
Ying Wu	Pfizer, New York, NY, United States	ying.wu3@pfizer.com	Clinical Groups	**Dermatology Group**
Pirkko Pussinen	Hospital District of Helsinki and Uusimaa, Helsinki, Finland	pirkko.pussinen@helsinki.fi	Clinical Groups	**Odontology Group**
Aino Salminen	Hospital District of Helsinki and Uusimaa, Helsinki, Finland	aino.m.salminen@helsinki.fi	Clinical Groups	**Odontology Group**
Tuula Salo	Hospital District of Helsinki and Uusimaa, Helsinki, Finland	tuula.salo@helsinki.fi	Clinical Groups	**Odontology Group**
David Rice	Hospital District of Helsinki and Uusimaa, Helsinki, Finland	david.rice@helsinki.fi	Clinical Groups	**Odontology Group**
Pekka Nieminen	Hospital District of Helsinki and Uusimaa, Helsinki, Finland	pekka.nieminen@helsinki.fi	Clinical Groups	**Odontology Group**
Ulla Palotie	Hospital District of Helsinki and Uusimaa, Helsinki, Finland	ulla.palotie@helsinki.fi	Clinical Groups	**Odontology Group**
Maria Siponen	Northern Savo Hospital District, Kuopio, Finland	maria.siponen@uef.fi	Clinical Groups	**Odontology Group**
Liisa Suominen	Northern Savo Hospital District, Kuopio, Finland	liisa.suominen@uef.fi	Clinical Groups	**Odontology Group**
Päivi Mäntylä	Northern Savo Hospital District, Kuopio, Finland	paivi.mantyla@uef.fi	Clinical Groups	**Odontology Group**
Ulvi Gursoy	Hospital District of Southwest Finland, Turku, Finland	ulvi.gursoy@utu.fi	Clinical Groups	**Odontology Group**
Vuokko Anttonen	Northern Ostrobothnia Hospital District, Oulu, Finland	vuokko.anttonen@oulu.fi	Clinical Groups	**Odontology Group**
Kirsi Sipilä	Research Unit of Oral Health Sciences Faculty of Medicine, University of Oulu, Oulu, Finland; Medical Research Center, Oulu, Oulu University Hospital and University of Oulu, Oulu, Finland	kirsi.sipila@oulu.fi	Clinical Groups	**Odontology Group**
Rion Pendergrass	Genentech, San Francisco, CA, United States	pendergrass.sarah@gene.com	Clinical Groups	**Odontology Group**
Hannele Laivuori	Institute for Molecular Medicine Finland, HiLIFE, University of Helsinki, Finland	hannele.laivuori@helsinki.fi	Clinical Groups	**Women's Health and Reproduction Group**
Venla Kurra	Pirkanmaa Hospital District, Tampere, Finland	venla.kurra@tuni.fi	Clinical Groups	**Women's Health and Reproduction Group**
Laura Kotaniemi-Talonen	Pirkanmaa Hospital District, Tampere, Finland	laura.kotaniemi-talonen@tuni.fi	Clinical Groups	**Women's Health and Reproduction Group**
Oskari Heikinheimo	Hospital District of Helsinki and Uusimaa, Helsinki, Finland	oskari.heikinheimo@helsinki.fi	Clinical Groups	**Women's Health and Reproduction Group**
Ilkka Kalliala	Hospital District of Helsinki and Uusimaa, Helsinki, Finland	ilkka.kalliala@hus.fi	Clinical Groups	**Women's Health and Reproduction Group**
Lauri Aaltonen	Hospital District of Helsinki and Uusimaa, Helsinki, Finland	lauri.aaltonen@helsinki.fi	Clinical Groups	**Women's Health and Reproduction Group**
Varpu Jokimaa	Hospital District of Southwest Finland, Turku, Finland	varpu.jokimaa@utu.fi	Clinical Groups	**Women's Health and Reproduction Group**
Johannes Kettunen	Northern Ostrobothnia Hospital District, Oulu, Finland	Johannes.Kettunen@oulu.fi	Clinical Groups	**Women's Health and Reproduction Group**
Marja Vääräsmäki	Northern Ostrobothnia Hospital District, Oulu, Finland	marja.vaarasmaki@oulu.fi	Clinical Groups	**Women's Health and Reproduction Group**
Outi Uimari	Northern Ostrobothnia Hospital District, Oulu, Finland	outi.uimari@oulu.fi	Clinical Groups	**Women's Health and Reproduction Group**
Laure Morin-Papunen	Northern Ostrobothnia Hospital District, Oulu, Finland	lmp@cc.oulu.fi	Clinical Groups	**Women's Health and Reproduction Group**
Maarit Niinimäki	Northern Ostrobothnia Hospital District, Oulu, Finland	maarit.niinimaki@oulu.fi	Clinical Groups	**Women's Health and Reproduction Group**
Terhi Piltonen	Northern Ostrobothnia Hospital District, Oulu, Finland	terhi.piltonen@oulu.fi	Clinical Groups	**Women's Health and Reproduction Group**
Katja Kivinen	Institute for Molecular Medicine Finland, HiLIFE, University of Helsinki, Finland	katja.kivinen@helsinki.fi	Clinical Groups	**Women's Health and Reproduction Group**
Elisabeth Widen	Institute for Molecular Medicine Finland, HiLIFE, University of Helsinki, Finland	elisabeth.widen@helsinki.fi	Clinical Groups	**Women's Health and Reproduction Group**
Taru Tukiainen	Institute for Molecular Medicine Finland, HiLIFE, University of Helsinki, Finland	taru.tukiainen@helsinki.fi	Clinical Groups	**Women's Health and Reproduction Group**
Mary Pat Reeve	Institute for Molecular Medicine Finland, HiLIFE, University of Helsinki, Finland	mary.reeve@helsinki.fi	Clinical Groups	**Women's Health and Reproduction Group**
Mark Daly	Institute for Molecular Medicine, Finland (FIMM), HiLIFE, University of Helsinki, Helsinki, Finland; Broad Institute of MIT and Harvard; Massachusetts General Hospital	mark.daly@helsinki.fi	Clinical Groups	**Women's Health and Reproduction Group**
Niko Välimäki	University of Helsinki, Helsinki, Finland	niko.valimaki@helsinki.fi	Clinical Groups	**Women's Health and Reproduction Group**
Eija Laakkonen	University of Jyväskylä, Jyväskylä, Finland	eija.k.laakkonen@jyu.fi	Clinical Groups	**Women's Health and Reproduction Group**
Jaakko Tyrmi	University of Oulu, Oulu, Finland / University of Tampere, Tampere, Finland	jaakko.tyrmi@oulu.fi	Clinical Groups	**Women's Health and Reproduction Group**
Heidi Silven	University of Oulu, Oulu, Finland	heidi.silven@student.oulu.fi	Clinical Groups	**Women's Health and Reproduction Group**
Eeva Sliz	University of Oulu, Oulu, Finland	eeva.sliz@oulu.fi	Clinical Groups	**Women's Health and Reproduction Group**
Riikka Arffman	University of Oulu, Oulu, Finland	riikka.arffman@oulu.fi	Clinical Groups	**Women's Health and Reproduction Group**
Susanna Savukoski	University of Oulu, Oulu, Finland	susanna.savukoski@oulu.fi	Clinical Groups	**Women's Health and Reproduction Group**
Triin Laisk	Estonian biobank, Tartu, Estonia	triin.laisk@ut.ee	Clinical Groups	**Women's Health and Reproduction Group**

Natalia Pujol	Estonian biobank, Tartu, Estonia	natalia.pujolgualdo@oulu.fi	Clinical Groups	Women's Health and Reproduction Group
Mengzhen Liu	Abbvie, Chicago, IL, United States	mengzhen.liu@abbvie.com	Clinical Groups	Women's Health and Reproduction Group
Bridget Riley-Gillis	Abbvie, Chicago, IL, United States	bridget.rileygillis@abbvie.com	Clinical Groups	Women's Health and Reproduction Group
Rion Pendergrass	Genentech, San Francisco, CA, United States	penders2@gene.com	Clinical Groups	Women's Health and Reproduction Group
Janet Kumar	GlaxoSmithKline, Collegeville, PA, United States	janet.x.kumar@gsk.com	Clinical Groups	Women's Health and Reproduction Group
Kirsi Auro	GlaxoSmithKline, Espoo, Finland	kirsi.m.auro@gsk.com	Clinical Groups	Women's Health and Reproduction Group
Iiris Hovatta	University of Helsinki, Finland	iiris.hovatta@helsinki.fi	Clinical Groups	Depression group
Chia-Yen Chen	Biogen, Cambridge, MA, United States	chiayen.chen@biogen.com	Clinical Groups	Depression group
Erkki Isometsä	Hospital District of Helsinki and Uusimaa, Helsinki, Finland	erkki.isometsa@hus.fi	Clinical Groups	Depression group
Kumar Veerapen	Broad Institute, Cambridge, MA, United States	veerapen@broadinstitute.org	Clinical Groups	Depression group
Hanna Ollila	Institute for Molecular Medicine Finland, HiLIFE, University of Helsinki, Finland	hanna.m.ollila@helsinki.fi	Clinical Groups	Depression group
Jaana Suvisaari	Finnish Institute for Health and Welfare (THL), Helsinki, Finland	jaana.suvisaari@thl.fi	Clinical Groups	Depression group
Thomas Damm Als	Aarhus University, Denmark	tda@biomed.au.dk	Clinical Groups	Depression group
Antti Mäkitie	Department of Otorhinolaryngology - Head and Neck Surgery, University of Helsinki and Helsinki University Hospital, Helsinki, Finland	antti.makitie@helsinki.fi	Clinical Groups	ENT (ear, nose and throath) Group
Argyro Bizaki-Vallaskangas	Pirkanmaa Hospital District, Tampere, Finland	argyro.bizaki-vallaskangas@tuni.fi	Clinical Groups	ENT (ear, nose and throath) Group
Sanna Toppila-Salmi	University of Helsinki, Finland	sanna.salmi@helsinki.fi	Clinical Groups	ENT (ear, nose and throath) Group
Tytti Willberg	Hospital District of Southwest Finland, Turku, Finland	tytti.willberg@tyks.fi	Clinical Groups	ENT (ear, nose and throath) Group
Elmo Saarentaus	Institute for Molecular Medicine Finland, HiLIFE, University of Helsinki, Finland	elmo.saarentaus@helsinki.fi	Clinical Groups	ENT (ear, nose and throath) Group
Antti Aarnisalo	Hospital District of Helsinki and Uusimaa, Helsinki, Finland	antti.aarnisalo@hus.fi	Clinical Groups	ENT (ear, nose and throath) Group
Eveliina Salminen	Hospital District of Helsinki and Uusimaa, Helsinki, Finland	eveliina.e.salminen@hus.fi	Clinical Groups	ENT (ear, nose and throath) Group
Elisa Rahikkala	Northern Ostrobothnia Hospital District, Oulu, Finland	elisa.rahikkala@ppshp.fi	Clinical Groups	ENT (ear, nose and throath) Group
Johannes Kettunen	Northern Ostrobothnia Hospital District, Oulu, Finland	johannes.kettunen@oulu.fi	Clinical Groups	ENT (ear, nose and throath) Group
Mitja Kurki	Institute for Molecular Medicine Finland, HiLIFE, University of Helsinki, Finland / Broad Institute, Cambridge, MA, United States	mkurki@broadinstitute.org	FinnGen Analysis working grou	FinnGen Analysis working group
Samuli Ripatti	Institute for Molecular Medicine Finland, HiLIFE, University of Helsinki, Finland	samuli.ripatti@helsinki.fi	FinnGen Analysis working grou	FinnGen Analysis working group
Mark Daly	Institute for Molecular Medicine, Finland (FIMM), HiLIFE, University of Helsinki, Helsinki, Finland; Broad Institute of MIT and Harvard; Massachusetts General Hospital	mark.daly@helsinki.fi	FinnGen Analysis working grou	FinnGen Analysis working group
Juha Karjalainen	Institute for Molecular Medicine Finland, HiLIFE, University of Helsinki, Finland	juha.karjalainen@helsinki.fi	FinnGen Analysis working grou	FinnGen Analysis working group
Aki Havulinna	Institute for Molecular Medicine Finland, HiLIFE, University of Helsinki, Finland	aki.havulinna@helsinki.fi	FinnGen Analysis working grou	FinnGen Analysis working group
Juha Mehtonen	Institute for Molecular Medicine Finland, HiLIFE, University of Helsinki, Finland	juha.mehtonen@helsinki.fi	FinnGen Analysis working grou	FinnGen Analysis working group
Priit Palta	Institute for Molecular Medicine Finland, HiLIFE, University of Helsinki, Finland	priit.palta@helsinki.fi	FinnGen Analysis working grou	FinnGen Analysis working group
Shabbeer Hassan	Institute for Molecular Medicine Finland, HiLIFE, University of Helsinki, Finland	shabbeer.hassan@helsinki.fi	FinnGen Analysis working grou	FinnGen Analysis working group
Pietro Della Briotta Parolo	Institute for Molecular Medicine Finland, HiLIFE, University of Helsinki, Finland	pietro.dellabriottaparolo@helsinki.fi	FinnGen Analysis working grou	FinnGen Analysis working group
Wei Zhou	Broad Institute, Cambridge, MA, United States	wzhou@broadinstitute.org	FinnGen Analysis working grou	FinnGen Analysis working group
Mutaamba Maasha	Broad Institute, Cambridge, MA, United States	mmaasha@broadinstitute.org	FinnGen Analysis working grou	FinnGen Analysis working group
Kumar Veerapen	Broad Institute, Cambridge, MA, United States	veerapen@broadinstitute.org	FinnGen Analysis working grou	FinnGen Analysis working group
Shabbeer Hassan	Institute for Molecular Medicine Finland, HiLIFE, University of Helsinki, Finland	shabbeer.hassan@helsinki.fi	FinnGen Analysis working grou	FinnGen Analysis working group
Susanna Lemmelä	Institute for Molecular Medicine Finland, HiLIFE, University of Helsinki, Finland	susanna.lemmela@helsinki.fi	FinnGen Analysis working grou	FinnGen Analysis working group
Manuel Rivas	University of Stanford, Stanford, CA, United States	mrivas@stanford.edu	FinnGen Analysis working grou	FinnGen Analysis working group
Mari E. Niemi	Institute for Molecular Medicine Finland, HiLIFE, University of Helsinki, Finland	mari.e.niemi@helsinki.fi	FinnGen Analysis working grou	FinnGen Analysis working group
Aarno Palotie	Institute for Molecular Medicine Finland, HiLIFE, University of Helsinki, Finland	aarno.palotie@helsinki.fi	FinnGen Analysis working grou	FinnGen Analysis working group
Aoxing Liu	Institute for Molecular Medicine Finland, HiLIFE, University of Helsinki, Finland	aoxing.liu@helsinki.fi	FinnGen Analysis working grou	FinnGen Analysis working group
Arto Lehisto	Institute for Molecular Medicine Finland, HiLIFE, University of Helsinki, Finland	arto.lehisto@helsinki.fi	FinnGen Analysis working grou	FinnGen Analysis working group
Andrea Ganna	Institute for Molecular Medicine Finland, HiLIFE, University of Helsinki, Finland	aganna@broadinstitute.org	FinnGen Analysis working grou	FinnGen Analysis working group
Vincent Llorens	Institute for Molecular Medicine Finland, HiLIFE, University of Helsinki, Finland	vincent.llorens@helsinki.fi	FinnGen Analysis working grou	FinnGen Analysis working group
Hannele Laivuori	Institute for Molecular Medicine Finland, HiLIFE, University of Helsinki, Finland	hannele.laivuori@helsinki.fi	FinnGen Analysis working grou	FinnGen Analysis working group
Taru Tukiainen	Institute for Molecular Medicine Finland, HiLIFE, University of Helsinki, Finland	taru.tukiainen@helsinki.fi	FinnGen Analysis working grou	FinnGen Analysis working group
Mary Pat Reeve	Institute for Molecular Medicine Finland, HiLIFE, University of Helsinki, Finland	mary.reeve@helsinki.fi	FinnGen Analysis working grou	FinnGen Analysis working group
Henrike Heyne	Institute for Molecular Medicine Finland, HiLIFE, University of Helsinki, Finland	hheyne@broadinstitute.org	FinnGen Analysis working grou	FinnGen Analysis working group
Nina Mars	Institute for Molecular Medicine Finland, HiLIFE, University of Helsinki, Finland	nina.mars@helsinki.fi	FinnGen Analysis working grou	FinnGen Analysis working group
Joel Rämö	Institute for Molecular Medicine Finland, HiLIFE, University of Helsinki, Finland	joel.ramo@helsinki.fi	FinnGen Analysis working grou	FinnGen Analysis working group
Elmo Saarentaus	Institute for Molecular Medicine Finland, HiLIFE, University of Helsinki, Finland	elmo.saarentaus@helsinki.fi	FinnGen Analysis working grou	FinnGen Analysis working group
Hanna Ollila	Institute for Molecular Medicine Finland, HiLIFE, University of Helsinki, Finland	hanna.m.ollila@helsinki.fi	FinnGen Analysis working grou	FinnGen Analysis working group
Rodos Rodosthenous	Institute for Molecular Medicine Finland, HiLIFE, University of Helsinki, Finland	rodos.rodosthenous@helsinki.fi	FinnGen Analysis working grou	FinnGen Analysis working group
Satu Strausz	Institute for Molecular Medicine Finland, HiLIFE, University of Helsinki, Finland	satu.strausz@helsinki.fi	FinnGen Analysis working grou	FinnGen Analysis working group
Tuula Palotie	University of Helsinki and Hospital District of Helsinki and Uusimaa, Helsinki, Finland	tuula.palotie@helsinki.fi	FinnGen Analysis working grou	FinnGen Analysis working group
Kimmo Palin	University of Helsinki, Helsinki, Finland	kimmo.palin@helsinki.fi	FinnGen Analysis working grou	FinnGen Analysis working group
Javier Garcia-Tabuenca	University of Tampere, Tampere, Finland	javier.graciatabuenca@tuni.fi	FinnGen Analysis working grou	FinnGen Analysis working group
Harri Siirtola	University of Tampere, Tampere, Finland	harri.siirtola@tuni.fi	FinnGen Analysis working grou	FinnGen Analysis working group
Tuomo Kiiskinen	Institute for Molecular Medicine Finland, HiLIFE, University of Helsinki, Finland	tuomo.kiiskinen@helsinki.fi	FinnGen Analysis working grou	FinnGen Analysis working group
Jiwoo Lee	Institute for Molecular Medicine Finland, HiLIFE, University of Helsinki, Finland; Broad Institute, Cambridge, MA, United States	jiwoo.lee@helsinki.fi	FinnGen Analysis working grou	FinnGen Analysis working group
Kristin Tsuo	Institute for Molecular Medicine Finland, HiLIFE, University of Helsinki, Finland; Broad Institute, Cambridge, MA, United States	kristintsuo@fas.harvard.edu	FinnGen Analysis working grou	FinnGen Analysis working group
Amanda Elliott	Institute for Molecular Medicine Finland, HiLIFE, University of Helsinki, Finland; Broad Institute, Cambridge, MA, USA and Massachusetts General Hospital, Boston, MA, USA	aelliott@broadinstitute.org	FinnGen Analysis working grou	FinnGen Analysis working group
Kati Kristiansson	THL Biobank / Finnish Institute for Health and Welfare (THL), Helsinki, Finland	kati.kristiansson@thl.fi	FinnGen Analysis working grou	FinnGen Analysis working group
Mikko Arvas	Finnish Red Cross Blood Service / Finnish Hematology Registry and Clinical Biobank, Helsinki, Finland	mikko.arvas@veripalvelu.fi	FinnGen Analysis working grou	FinnGen Analysis working group
Kati Hyvärinen	Finnish Red Cross Blood Service, Helsinki, Finland	kati.hyvarinen@veripalvelu.fi	FinnGen Analysis working grou	FinnGen Analysis working group
Jarmo Ritari	Finnish Red Cross Blood Service, Helsinki, Finland	jarmo.ritari@veripalvelu.fi	FinnGen Analysis working grou	FinnGen Analysis working group
Olli Carpén	Helsinki Biobank / Helsinki University and Hospital District of Helsinki and Uusimaa, Helsinki	olli.carpen@helsinki.fi	FinnGen Analysis working grou	FinnGen Analysis working group
Johannes Kettunen	Northern Finland Biobank Borealis / University of Oulu / Northern Ostrobothnia Hospital District, Oulu, Finland	johannes.kettunen@oulu.fi	FinnGen Analysis working grou	FinnGen Analysis working group
Katri Pylkäs	University of Oulu, Oulu, Finland	katri.pylkas@oulu.fi	FinnGen Analysis working grou	FinnGen Analysis working group
Eeva Sliz	University of Oulu, Oulu, Finland	eeva.sliz@oulu.fi	FinnGen Analysis working grou	FinnGen Analysis working group
Minna Karjalainen	University of Oulu, Oulu, Finland	minna.k.karjalainen@oulu.fi	FinnGen Analysis working grou	FinnGen Analysis working group
Tuomo Mantere	Northern Finland Biobank Borealis / University of Oulu / Northern Ostrobothnia Hospital District, Oulu, Finland	tuomo.mantere@oulu.fi	FinnGen Analysis working grou	FinnGen Analysis working group
Eeva Kangasniemi	Finnish Clinical Biobank Tampere / University of Tampere / Pirkanmaa Hospital District, Tampere, Finland	eeva.kangasniemi@pshp.fi	FinnGen Analysis working grou	FinnGen Analysis working group
Sami Heikkinen	University of Eastern Finland, Kuopio, Finland	sami.heikkinen@uef.fi	FinnGen Analysis working grou	FinnGen Analysis working group
Arto Mannermaa	Biobank of Eastern Finland / University of Eastern Finland / Northern Savo Hospital District, Kuopio, Finland	arto.mannermaa@uef.fi	FinnGen Analysis working grou	FinnGen Analysis working group
Eija Laakkonen	University of Jyväskylä, Jyväskylä, Finland	eija.k.laakkonen@jyu.fi	FinnGen Analysis working grou	FinnGen Analysis working group
Nina Pitkänen	Auria Biobank / University of Turku / Hospital District of Southwest Finland, Turku, Finland	Niina.Pitkanen@tyks.fi	FinnGen Analysis working grou	FinnGen Analysis working group
Samuel Lessard	Translational Sciences, Sanofi R&D, Framingham, MA, USA	samuel.lessard@sanofi.com	FinnGen Analysis working grou	FinnGen Analysis working group
Clément Chatelain	Translational Sciences, Sanofi R&D, Framingham, MA, USA	clement.chatelain@sanofi.com	FinnGen Analysis working grou	FinnGen Analysis working group
Perttu Terho	Auria Biobank / University of Turku / Hospital District of Southwest Finland, Turku, Finland	perttu.terho@tyks.fi	Biobank directors	Biobank directors
Sirpa Soini	THL Biobank / Finnish Institute for Health and Welfare (THL), Helsinki, Finland	sirpa.soini@thl.fi	Biobank directors	Biobank directors
Jukka Partanen	Finnish Red Cross Blood Service / Finnish Hematology Registry and Clinical Biobank, Helsinki, Finland	jukka.partanen@veripalvelu.fi	Biobank directors	Biobank directors
Eero Punkka	Helsinki Biobank / Helsinki University and Hospital District of Helsinki and Uusimaa, Helsinki	eero.punkka@hus.fi	Biobank directors	Biobank directors
Raisa Serpi	Northern Finland Biobank Borealis / University of Oulu / Northern Ostrobothnia Hospital District, Oulu, Finland	raisa.serpi@ppshp.fi	Biobank directors	Biobank directors
Sanna Siltanen	Finnish Clinical Biobank Tampere / University of Tampere / Pirkanmaa Hospital District, Tampere, Finland	sanna.siltanen@pshp.fi	Biobank directors	Biobank directors
Veli-Matti Kosma	Biobank of Eastern Finland / University of Eastern Finland / Northern Savo Hospital District, Kuopio, Finland	veli-matti.kosma@uef.fi	Biobank directors	Biobank directors
Teijo Kuopio	Central Finland Biobank / University of Jyväskylä / Central Finland Health Care District, Jyväskylä, Finland	teijo.kuopio@ksshp.fi	Biobank directors	Biobank directors
Anu Jalanko	Institute for Molecular Medicine Finland, HiLIFE, University of Helsinki, Finland	anu.jalanko@helsinki.fi	FinnGen Teams	Administration
Huei-Yi Shen	Institute for Molecular Medicine Finland, HiLIFE, University of Helsinki, Finland	huei-yi.shen@helsinki.fi	FinnGen Teams	Administration
Risto Kajanne	Institute for Molecular Medicine Finland, HiLIFE, University of Helsinki, Finland	risto.kajanne@helsinki.fi	FinnGen Teams	Administration
Mervi Aavikko	Institute for Molecular Medicine Finland, HiLIFE, University of Helsinki, Finland	mervi.aavikko@helsinki.fi	FinnGen Teams	Administration
Mitja Kurki	Institute for Molecular Medicine Finland, HiLIFE, University of Helsinki, Finland / Broad Institute, Cambridge, MA, United States	mkurki@broadinstitute.org	FinnGen Teams	Analysis
Juha Karjalainen	Institute for Molecular Medicine Finland, HiLIFE, University of Helsinki, Finland	juha.karjalainen@helsinki.fi	FinnGen Teams	Analysis
Pietro Della Briotta Parolo	Institute for Molecular Medicine Finland, HiLIFE, University of Helsinki, Finland	pietro.dellabriottaparolo@helsinki.fi	FinnGen Teams	Analysis

Arto Lehisto	Institute for Molecular Medicine Finland, HiLIFE, University of Helsinki, Finland	arto.lehisto@helsinki.fi	FinnGen Teams	**Analysis**
Juha Mehtonen	Institute for Molecular Medicine Finland, HiLIFE, University of Helsinki, Finland	juha.mehtonen@helsinki.fi	FinnGen Teams	**Analysis**
Wei Zhou	Broad Institute, Cambridge, MA, United States	wzhou@broadinstitute.org	FinnGen Teams	**Analysis**
Masahiro Kanai	Broad Institute, Cambridge, MA, United States	mkanai@broadinstitute.org	FinnGen Teams	**Analysis**
Mutaamba Maasha	Broad Institute, Cambridge, MA, United States	mmaasha@broadinstitute.org	FinnGen Teams	**Analysis**
Kumar Veerapen	Broad Institute, Cambridge, MA, United States	veerapen@broadinstitute.org	FinnGen Teams	**Analysis**
Hannele Laivuori	Institute for Molecular Medicine Finland, HiLIFE, University of Helsinki, Finland	hannele.laivuori@helsinki.fi	FinnGen Teams	**Clinical Endpoint Development**
Aki Havulinna	Institute for Molecular Medicine Finland, HiLIFE, University of Helsinki, Finland	aki.havulinna@helsinki.fi	FinnGen Teams	**Clinical Endpoint Development**
Susanna Lemmelä	Institute for Molecular Medicine Finland, HiLIFE, University of Helsinki, Finland	susanna.lemmela@helsinki.fi	FinnGen Teams	**Clinical Endpoint Development**
Tuomo Kiiskinen	Institute for Molecular Medicine Finland, HiLIFE, University of Helsinki, Finland	tuomo.kiiskinen@helsinki.fi	FinnGen Teams	**Clinical Endpoint Development**
L. Elisa Lahtela	Institute for Molecular Medicine Finland, HiLIFE, University of Helsinki, Finland	laura.lahtela@helsinki.fi	FinnGen Teams	**Clinical Endpoint Development**
Mari Kaunisto	Institute for Molecular Medicine Finland, HiLIFE, University of Helsinki, Finland	mari.kaunisto@helsinki.fi	FinnGen Teams	**Communication**
Elina Kilpeläinen	Institute for Molecular Medicine Finland, HiLIFE, University of Helsinki, Finland	elina.kilpelainen@helsinki.fi	FinnGen Teams	**E-Science**
Timo P. Sipilä	Institute for Molecular Medicine Finland, HiLIFE, University of Helsinki, Finland	timo.p.sipila@helsinki.fi	FinnGen Teams	**E-Science**
Oluwaseun Alexander Dada	Institute for Molecular Medicine Finland, HiLIFE, University of Helsinki, Finland	alexander.dada@helsinki.fi	FinnGen Teams	**E-Science**
Awaisa Ghazal	Institute for Molecular Medicine Finland, HiLIFE, University of Helsinki, Finland	awaisa.ghazal@helsinki.fi	FinnGen Teams	**E-Science**
Anastasia Shcherban	Institute for Molecular Medicine Finland, HiLIFE, University of Helsinki, Finland	anastasia.shcherban@helsinki.fi	FinnGen Teams	**E-Science**
Rigbe Weldatsadik	Institute for Molecular Medicine Finland, HiLIFE, University of Helsinki, Finland	rigbe.weldatsadik@helsinki.fi	FinnGen Teams	**E-Science**
Kati Donner	Institute for Molecular Medicine Finland, HiLIFE, University of Helsinki, Finland	kati.donner@helsinki.fi	FinnGen Teams	**Genotyping**
Timo P. Sipilä	Institute for Molecular Medicine Finland, HiLIFE, University of Helsinki, Finland	timo.p.sipila@helsinki.fi	FinnGen Teams	**Genotyping**
Anu Loukola	Helsinki Biobank / Helsinki University and Hospital District of Helsinki and Uusimaa, Helsinki	anu.loukola@hus.fi	FinnGen Teams	**Sample Collection Coordination**
Päivi Laiho	THL Biobank / Finnish Institute for Health and Welfare (THL), Helsinki, Finland	paivi.laiho@thl.fi	FinnGen Teams	**Sample Logistics**
Tuuli Sistonen	THL Biobank / Finnish Institute for Health and Welfare (THL), Helsinki, Finland	tuuli.sistonen@thl.fi	FinnGen Teams	**Sample Logistics**
Essi Kaiharju	THL Biobank / Finnish Institute for Health and Welfare (THL), Helsinki, Finland	essi.kaiharju@thl.fi	FinnGen Teams	**Sample Logistics**
Markku Laukkanen	THL Biobank / Finnish Institute for Health and Welfare (THL), Helsinki, Finland	markku.laukkanen@thl.fi	FinnGen Teams	**Sample Logistics**
Elina Järvensivu	THL Biobank / Finnish Institute for Health and Welfare (THL), Helsinki, Finland	elina.jarvensivu@thl.fi	FinnGen Teams	**Sample Logistics**
Sini Lähteenmäki	THL Biobank / Finnish Institute for Health and Welfare (THL), Helsinki, Finland	sini.lahteenmaki@thl.fi	FinnGen Teams	**Sample Logistics**
Lotta Männikkö	THL Biobank / Finnish Institute for Health and Welfare (THL), Helsinki, Finland	lotta.mannikko@thl.fi	FinnGen Teams	**Sample Logistics**
Regis Wong	THL Biobank / Finnish Institute for Health and Welfare (THL), Helsinki, Finland	regis.wong@thl.fi	FinnGen Teams	**Sample Logistics**
Minna Brunfeldt	THL Biobank / Finnish Institute for Health and Welfare (THL), Helsinki, Finland	minna.brunfeldt@thl.fi	FinnGen Teams	**Registry Data Operations**
Hannele Mattsson	THL Biobank / Finnish Institute for Health and Welfare (THL), Helsinki, Finland	hannele.mattsson@thl.fi	FinnGen Teams	**Registry Data Operations**
Kati Kristiansson	THL Biobank / Finnish Institute for Health and Welfare (THL), Helsinki, Finland	kati.kristiansson@thl.fi	FinnGen Teams	**Registry Data Operations**
Susanna Lemmelä	Institute for Molecular Medicine Finland, HiLIFE, University of Helsinki, Finland	susanna.lemmela@helsinki.fi	FinnGen Teams	**Registry Data Operations**
Sami Koskelainen	THL Biobank / Finnish Institute for Health and Welfare (THL), Helsinki, Finland	sami.koskelainen@thl.fi	FinnGen Teams	**Registry Data Operations**
Tero Hiekkalinna	THL Biobank / Finnish Institute for Health and Welfare (THL), Helsinki, Finland	tero.hiekkalinna@helsinki.fi	FinnGen Teams	**Registry Data Operations**
Teemu Paajanen	THL Biobank / Finnish Institute for Health and Welfare (THL), Helsinki, Finland	teemu.paajanen@thl.fi	FinnGen Teams	**Registry Data Operations**
Priit Palta	Institute for Molecular Medicine Finland, HiLIFE, University of Helsinki, Finland	priit.palta@helsinki.fi	FinnGen Teams	**Sequencing Informatics**
Kalle Pärn	Institute for Molecular Medicine Finland, HiLIFE, University of Helsinki, Finland	kalle.parn@helsinki.fi	FinnGen Teams	**Sequencing Informatics**
Mart Kals	Institute for Molecular Medicine Finland, HiLIFE, University of Helsinki, Finland	mart.kals@helsinki.fi	FinnGen Teams	**Sequencing Informatics**
Shuang Luo	Institute for Molecular Medicine Finland, HiLIFE, University of Helsinki, Finland	shuang.luo@helsinki.fi	FinnGen Teams	**Sequencing Informatics**
Vishal Sinha	Institute for Molecular Medicine Finland, HiLIFE, University of Helsinki, Finland	vishal.sinha@helsinki.fi	FinnGen Teams	**Sequencing Informatics**
Tarja Laitinen	Pirkanmaa Hospital District, Tampere, Finland	tarja.laitinen@pshp.fi	FinnGen Teams	**Trajectory**
Mary Pat Reeve	Institute for Molecular Medicine Finland, HiLIFE, University of Helsinki, Finland	mary.reeve@helsinki.fi	FinnGen Teams	**Trajectory**
Marianna Niemi	University of Tampere, Tampere, Finland	marianna.niemi@tuni.fi	FinnGen Teams	**Trajectory**
Kumar Veerapen	Broad Institute, Cambridge, MA, United States	veerapen@broadinstitute.org	FinnGen Teams	**Trajectory**
Harri Siirtola	University of Tampere, Tampere, Finland	harri.siirtola@tuni.fi	FinnGen Teams	**Trajectory**
Javier Gracia-Tabuenca	University of Tampere, Tampere, Finland	javier.graciatabuenca@tuni.fi	FinnGen Teams	**Trajectory**
Mika Helminen	University of Tampere, Tampere, Finland	mika.helminen@tuni.fi	FinnGen Teams	**Trajectory**
Tiina Luukkaala	University of Tampere, Tampere, Finland	tiina.luukkaala@tuni.fi	FinnGen Teams	**Trajectory**
Iida Vähätalo	University of Tampere, Tampere, Finland	iida.vahatalo@epshp.fi	FinnGen Teams	**Trajectory**
Jyrki Pitkänen	Institute for Molecular Medicine Finland, HiLIFE, University of Helsinki, Finland	jyrki.pitkanen@helsinki.fi	FinnGen Teams	**Data protection officer**
Marco Hautalahti		marco.hautalahti@finbb.fi	FinnGen Teams	**FINBB - Finnish biobank cooperative**
Johanna Mäkelä		johanna.makela@finbb.fi	FinnGen Teams	**FINBB - Finnish biobank cooperative**
Sarah Smith		sarah.smith@finbb.fi	FinnGen Teams	**FINBB - Finnish biobank cooperative**
Tom Southerington		tom.southerington@finbb.fi	FinnGen Teams	**FINBB - Finnish biobank cooperative**
